# Inferring associations among behavior, hormonal, neurotransmitter and IgA responses of dairy cows housed in automatic milking systems

**DOI:** 10.1016/j.vas.2026.100857

**Published:** 2026-09-05

**Authors:** Laura Breitmeier, Kerstin Brügemann, Gerhard Schuler, Larissa Behren, Julia Stuhlträger, Frank Rosner, Hermann Swalve, Sven König, Katharina May

**Affiliations:** a Institute of Animal Breeding and Genetics, Justus-Liebig-University Gießen, 35390 Gießen, Germany; b Veterinary Clinic for Reproductive Medicine and Neonatology, Faculty of Veterinary Medicine, Justus-Liebig-University Gießen, 35392 Giessen, Germany; c Institute of Agricultural and Nutritional Sciences, Group Animal Breeding, Martin Luther University Halle-Wittenberg, 06120 Halle, Germany; d Institute for Parasitology, Faculty of Veterinary Medicine, Leipzig University, 04103 Leipzig, Germany

**Keywords:** Behavior tests, Physiological stress parameters, Stress biomarkers, Technical environment

## Abstract

This study investigated associations between the hormone cortisol, the neurotransmitters dopamine and serotonin, immunoglobulin A (IgA), heart rate (HR), rectal temperature (RT) and cow behavior pattern in relation to production stress, stress around calving, human handling and adaptation to the milking robot system. Blood samples of 315 Holstein-Friesian cows from five farms with AMS were taken within 14 d after calving (blood sampling date 1 (= BS1) to assess physiological responses during the early postpartum period, followed by human behavioral assessments at blood sampling date 2 (= BS2). Indicators for cow reactions to human handling at BS2 included measurements for the avoidance distance (AD) and scores for the tolerance to tactile interaction (TTI). Dopamine, serotonin and IgA were measured by ELISA tests, and cortisol concentrations were determined by radioimmunoassay. HR and RT as physiological stress indicators were recorded before blood sampling. Associations between blood and physiological biomarkers under different stressors were inferred via generalized linear mixed model applications. In model group 1, analyzing the effects of blood and physiological biomarkers at BS1 on the same biomarkers at BS1, serotonin significantly (*P* < 0.001) influenced dopamine, and vice versa. Model group 2, examining the effects of behavioral traits on blood and physiological biomarkers at BS2, revealed a significant effect of TTI (*P* < 0.001) on HR. In model group 3, assessing the effects of biomarkers at BS1 on the same biomarkers at BS2, dopamine, serotonin, IgA and HR at BS1 significantly (*P* < 0.001) predicted the corresponding biomarker at BS2. In model group 4, effects of daily milk yield on blood and physiological biomarkers at BS2, were non-significant (*P* > 0.05). In conclusion and from a practical farming perspective, AMS behavioral indicators showed diverse patterns in relation to stress biomarkers, suggesting an index trait for behavioral assessment in a technical environment.

## Introduction

Cattle behavior is controlled by a complex interplay of physiological processes, with hormones and neurotransmitters playing pivotal roles. Cortisol, which is secreted via the hypothalamic-pituitary-adrenal (**HPA**) axis, is a key hormone involved in behavior and stress-related responses. Chronic stress activates the HPA axis, leading to increased secretion of cortisol. Persistently elevated cortisol levels can impair immune functions and disrupt metabolic processes, thereby weakening various physiological systems. Over time, this dysregulation may contribute to the development of clinical diseases such as mastitis (Hopster et al., 1998) and fertility disorders (Dobson and Smith, 2000; von Borell et al., 2007). The risk of disease can be influenced directly by the activation of the HPA axis and its immunomodulatory effects, and indirectly by neuroendocrine biomarkers altering behavior patterns. Cortisol levels are also influenced by acute stressors, with short-term elevations reported in more temperamental cows (Hemsworth et al., 2011; Cooke et al., 2019; Nejad et al., 2021) and causing unfavorable effects on milk yield (Burnett et al., 2015). Thus, neurochemical imbalances of specific hormones or neurotransmitters can trigger behavioral changes which, in turn, contribute to economic losses due to increased disease susceptibility and impairments in milk production.

The neurotransmitters dopamine and serotonin, which are regulated by the HPA axis, play an essential role in physiological processes during lactation in dairy cows. Dopamine influences endocrine regulation via the dopamine D2 receptor, located on bovine chromosome 15. This receptor modulates the secretion of prolactin, a hormone directly involved in lactation (Campbell, 2012). Dopamine exerts an inhibitory effect on prolactin release, thereby reducing its plasma concentrations and consequently leading to decreased milk production (Lacasse et al., 2016). Serotonin is important for regulating calcium metabolism and homeostasis in lactating cows (Laporta et al., 2015). Low plasma serotonin concentrations on the first day of lactation were associated with an increased risk for ketosis during the first ten days after calving (Laporta et al., 2013). In addition, dopamine and serotonin are intimately involved in thermoregulatory signaling in the hypothalamus. Thus, these neurotransmitters not only modulate responses to stress but also influence physiological parameters such as body temperature and heart rate (**HR**). Raised HR and rectal temperature (**RT**) were observed in more temperamental cattle (Wenzel et al., 2003; Burdick et al., 2010; Friedrich et al., 2015), indicating a clear link between neurochemical control and behavior traits.

Cortisol, dopamine and serotonin are widely used indicators for cattle welfare and behavior (Friedrich et al., 2015; Chen et al., 2020). In the context of stress, welfare, and behavior, cortisol primarily reflects activation of the stress axis (Brown and Voslo, 2017), serotonin is associated with stress regulation, affective state and welfare-related processes (Cooke et al. 2023), and dopamine is involved in motivation, reward processing and stress-related behavioral modulation (Salamone and Correa, 2024). Research on further potential biomarkers in such context is limited. Staley et al. (2018) pointed out the role of immunoglobulin A (**IgA**) as a biomarker for psychological stress. Serum, salivary or fecal IgA concentrations changed in various pet and livestock species due to social or production environment alterations. Royo et al. (2005) reported increased secretory IgA in pigs during the first 12 days of chronic isolation stress. In contrast, in dogs, stable IgA patterns were observed after separations from conspecifics (Walker et al., 2014). Acute stress was associated with reduced secretory IgA in rats and dogs (Guhad and Hau, 1996; Kikkawa et al., 2003). Secretory IgA played a central role in protecting mucosal surfaces by preventing translocation of pathogens (Snoeck et al., 2006). Low IgA concentrations were reported during bacterial proliferation and inflammation or diarrhea (Cunningham-Rundles, 2001), additionally suggesting IgA as a biomarker for health.

Neurotransmitters and IgA have been analyzed in relation to milk secretion or stress in beef cattle (Mao et al., 2022; Caceres et al., 2023). Stress is manifested physiologically as well as through changes in milking behavior. Automatic milking systems (**AMS**) generate a technical environment to quantify behavioral responses to stress in dairy cows. Milking robots automatically record key indicators for assessing milking behavior, including milking duration, start and end times, intervals between milkings, milking frequency, milking speed and milk yield (Solano et al., 2022). Other characteristics, such as the number of incomplete milkings, and aggressive behavior during milking, provided deep insights into cow temperament pattern (Wethal and Heringstad, 2020).

Overall, conducted research indicates a complex interplay among blood biomarkers and cow behavior responses in relation to various stressors, to technical systems adaptations and to milk yield. In consequence, the objective of this study was to infer associations between cow response traits from specific stress situations (after calving, behavior assessments, milk production) at specific dates, i.e., i) associations among blood and physiological biomarkers after calving, ii) associations among blood biomarkers, physiological biomarkers and behavior traits after human handling, iii) interactions among these biomarkers after calving and human handling, and iv) effects of milk production and milking behavior on stress-related biomarkers.

## Materials and methods

### Ethical approval

Data sampling was performed under protocols approved by the state government of North Rhine-Westphalia, Germany, and in accordance with the German Animal Welfare Act (permit number: 81-02.04.40. 2023.VG082).

### Cows

The study population included 315 Holstein-Friesian (**HF**) dairy cows from five commercial dairy farms located in the German federal state of North Rhine-Westphalia. The cows were in their first (*N* = 129), second (*N* = 103) or third (*N* = 83) parity. Herd sizes ranged from 125 to 260 lactating cows. All farms participated in the 'Kuhvision' project (Reents et al., 2017) implying detailed health trait monitoring and cow genotyping supervised by the breeding organization. The farms were equipped with self-locking feeding gates to measure responses of human handling, and with **AMS** to generate dense behavior patterns in a technical environment. Two farms milked their cows with Delaval and three farms with Lely robots. We have selected the milking robot herds for this study based on the implementation of voluntary cow traffic, implying a same study design for both robot systems Delaval and Lely.

In a preliminary pilot study, 10 cows from one farm were randomly selected to represent a specific stress group to measure concentrations of cortisol, dopamine, serotonin and IgA in a particularly stressful situation, i.e., during fixation in a claw care crush after claw trimming. The data of the stress group served as a `ence for biomarkers under extreme stressful conditions, because of the lack of established physiological reference ranges for stress biomarkers in dairy cows. The respective statistical analyses, preliminary results from the small initial dataset, and interpretations of results from this pilot study are outlined in supplementary material S1.

### Blood sampling and storage

For the determination of the sample size for mixed model analyses, we performed an a priori power analysis with an effect size of *f* = 0.25 (according to Cohen (2013) for standardized differences of means), a significance level of 0.05 for the differences of means and a power of 0.80 in the G*Power program (version 3.1.9.7, University of Düsseldorf, Düsseldorf, Germany), suggesting 324 cows. Before blood sampling, the cows were visually assessed for any obvious clinical abnormalities, and the farmers were asked about any known diseases or health problems. Cows presenting evident abnormalities during this screening were excluded from the trial, and cows in heat were excluded. The final dataset for trait recording comprised 315 cows.

**Fig. 1:**
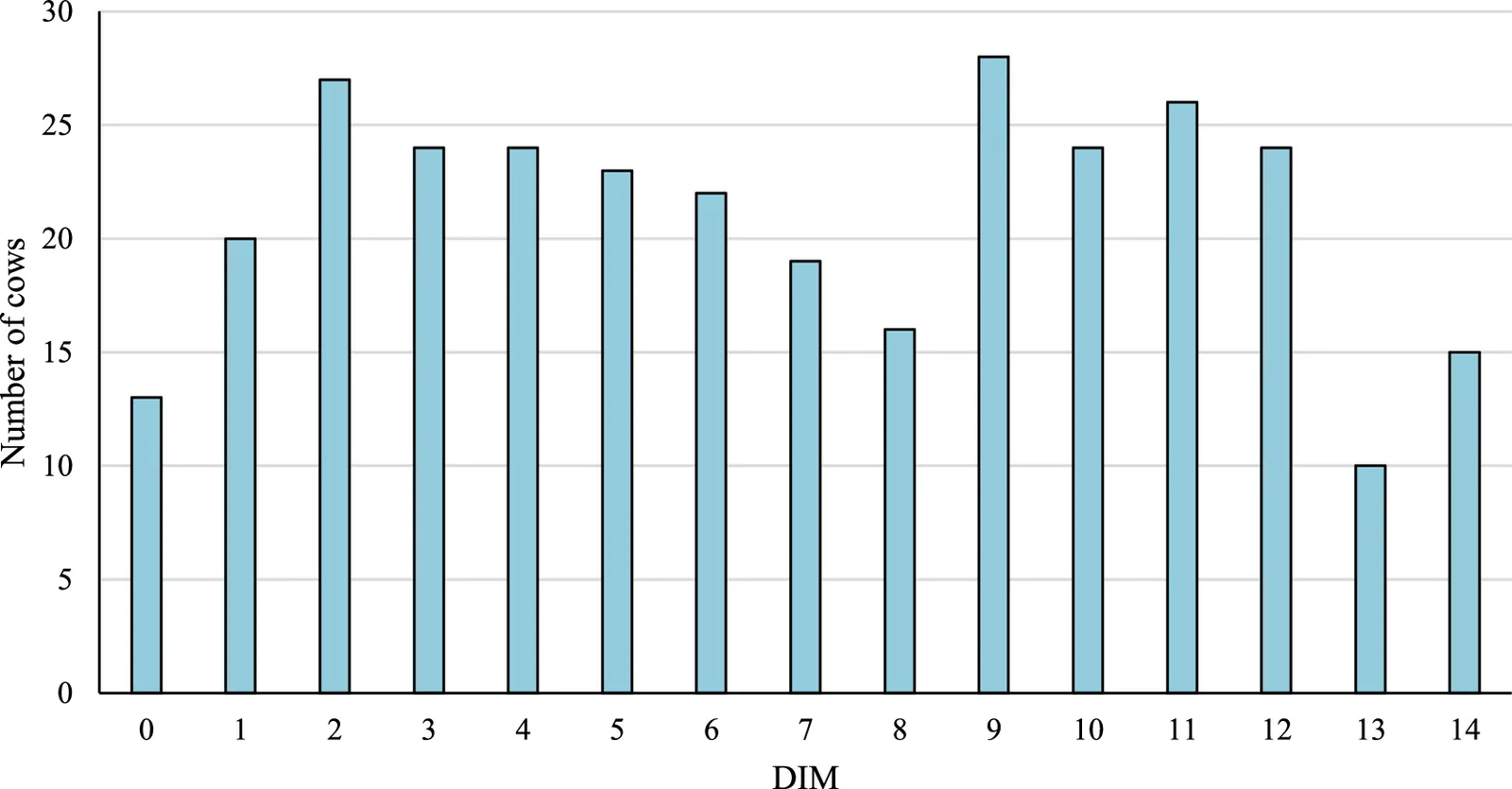
Distribution of the 315 cows included in blood sampling 1 (BS1) by days in milk (DIM) covering the 0–14 d postpartum period.

Each farm was visited in intervals of two weeks from the end of March 2024 to the beginning of November 2024 (blood sampling 1 = **BS1**). Blood samples at BS1 were taken from the 315 cows in the narrow period of 14 d postpartum, with 13 cows sampled on day 0 (calving) and 15 cows sampled on day 14 postpartum (Fig. 1). The cows were fixed in self-locking feeding gates for blood sampling and measuring HR and RT immediately before taking the blood. At the end of January 2025, a second blood sample (blood sampling 2 = **BS2**) was taken from the 315 cows (i.e., the same cows from BS1) after a behavior assessment to test cow reactions as response to human handling. At BS2, first the cows were fixed in the self-locking gates to record the avoidance distance (**AD**) and tolerance to tactile interaction (**TTI**). Directly after the behavior assessment, HR and RT were measured directly before blood sampling at BS2. The mean time interval between BS1 and BS2 comprised 181 days.

**Fig. 2:**
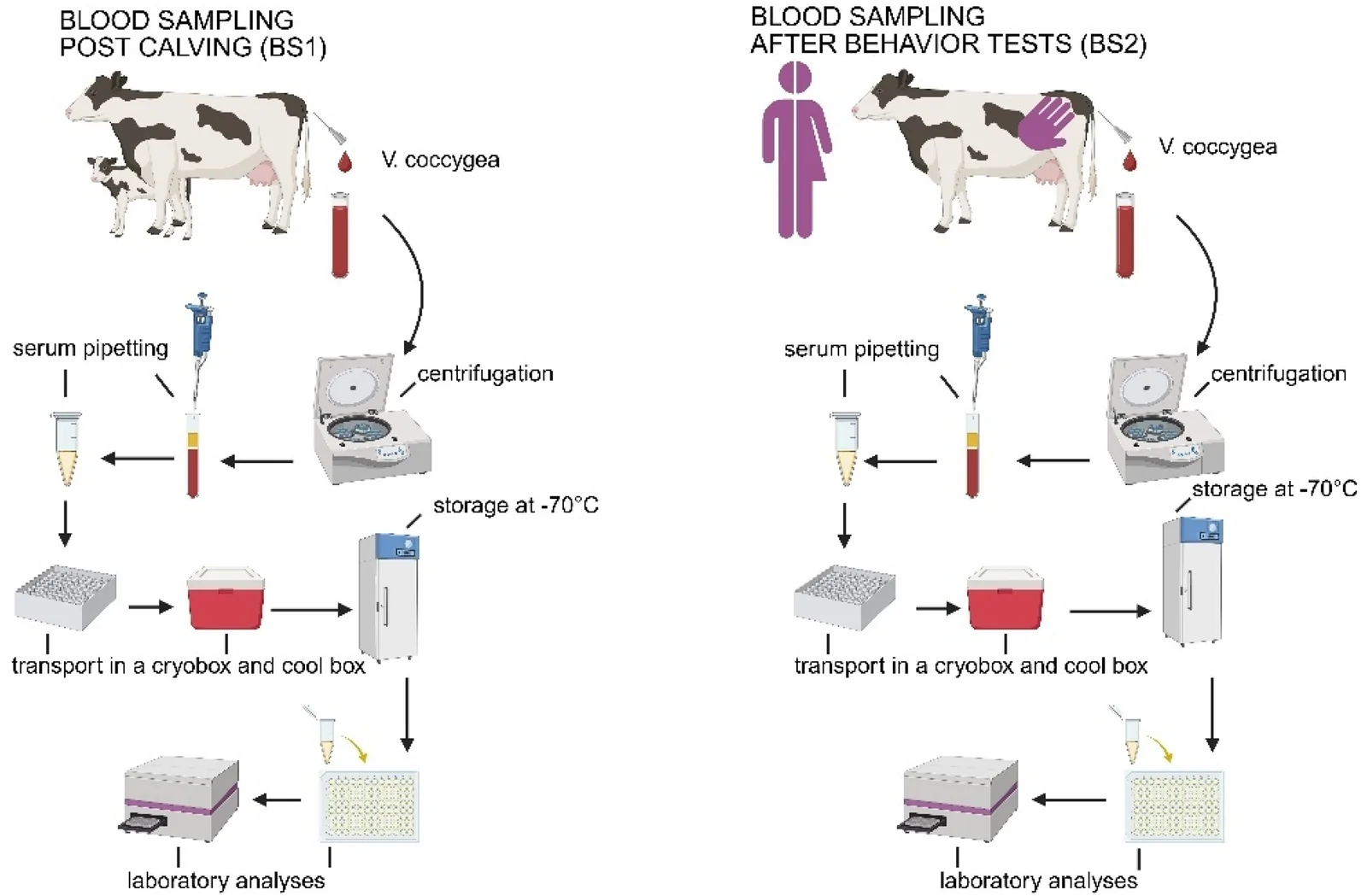
Schematic representation of the experimental workflow for sampling times and blood collection for biomarker assessment. Created in BioRender. Breitmeier, L. (2025) https://BioRender.com/5mww7iv.

Blood sampling was performed at approximately the same time of day between 10:00 and 12:00 across herds, since biomarker concentrations may be influenced by circadian variation. Blood was taken from the coccygeal vein (*vena coccygea*) by gently elevating the tail to facilitate venipuncture. A 1.6 × 25 mm LUER-LOCK needle and a 9 ml serum monovette (SARSTEDT ®, Nümbrecht, Germany) were used for sample collection. Immediately after collection, the blood samples were centrifuged at 970 × g for 30 min using a benchtop centrifuge (EBA 200 S, GEYER, Germany). The resulting serum was aliquoted into 1 ml microcentrifuge tubes (Eppendorf, Hamburg, Germany). Samples were transported in a portable cooling box to the molecular laboratory of the Institute of Animal Breeding and Genetics, Gießen, and stored at −70 °C until blood biomarker determinations. A schematic overview of the experimental workflow is illustrated in Fig. 2.

### Blood biomarkers

***Cortisol.*** The cortisol concentration was measured with a validated in-house radioimmunoassay (**RIA**) as previously described by Schuler et al. (2014) and Westhoff et al. (2022). A modified extraction protocol was used for sample preparation. The biological matrix was initially diluted in 40 mmol/L glutamic acid solution and subsequently heated under boiling conditions for 30 min (Donohue et al., 1975). Hormone quantification was carried out using a tritium-labeled tracer, and separation of free from antibody-bound cortisol was achieved via activated charcoal adsorption. The antiserum employed was directed against cortisol-3-carboxymethyl-oxime conjugated to bovine serum albumin. Cross-reactivity with other steroids-including androstenedione, 5α-dihydrotestosterone (5α-DHT), estradiol-17β, pregnenolone, progesterone, testosterone and corticosterone were <0.01 % in all cases. The intra- and inter-assay coefficients of variation were 4.4 % and 9.8 %, respectively. The assay detection limit was 0.36 ng/mL (equivalent to 1 nmol/L). All samples were analyzed in duplicate.

***Dopamine.*** The dopamine concentration was determined using a commercial ELISA Kit, (AssayGenie, competitive bovine dopamine enzyme-linked immunosorbent assay, BOEB1219, Dublin, Irleand) with a detection range of 1.56–100 ng/mL. The intra- and inter-assay variation coefficients were 6.1 % and 10.2 %, respectively. The ELISA was performed following the manufacturer protocol. A volume of 50 µL of sample standard and “Detection Reagent A” was added to each well of the microplate, followed by incubation for 60 min at 37 °C. The plate was washed three times, and then 100 µL of “Detection Reagent B” was added to each well and incubated for an additional 60 min at 37 °C. After five washes, 90 µL of substrate solution was added to each well, and the plate was incubated in the dark for 10 to 20 min. The reaction was stopped by adding 50 µL of stop solution per well. Absorbance was measured at 450 nm using a microplate reader (DYNEX MAGELLAN BIOSCIENCES, Chantilly, Virginia). According to the manufacturer, the analytical sensitivity of the assay was 0.49 ng/mL and is validated for the measurement of dopamine in bovine serum, plasma, and cell culture supernatants. Dilution linearity in bovine serum samples across serial dilutions (1:2 to 1:16) ranged from 89 % to 127 %, indicating acceptable assay parallelism.

***Serotonin.*** The serotonin concentration was measured using a serotonin enzyme-linked immunosorbent assay (**ELISA**) Kit (AssayGenie, competitive bovine serotonin (5HT) enzyme-linked immunosorbent assay, BOEB1217, Dublin, Ireland) with a detection range of 0.78–50 ng/mL. The intra- and inter-assay variation coefficients were 4.7 % and 7.7 %, respectively. To obtain measurable concentration within the standard range of the assay, samples were diluted 1:20. The ELISA was performed according to the manufacturer protocol and following the same procedure as described for dopamine. The manufacturer protocol indicated an analytical sensitivity of the assay of 0.35 ng/mL. Accordingly, in initial experiments, various sample dilutions were examined to confirm their suitability within the assay's working range prior to the final selection of the 1:20 dilution for all subsequent analyses.

***Immunoglobulin A.*** The IgA concentration was measured using a commercial ELISA Kit (Bethyl Laboratories, sandwich bovine, enzyme-linked immunosorbent assay, E11-131-220330, Montgomery, Texas, USA) with a detection range of 1.37 – 1000 ng/ml. The intra- and inter-assay variation coefficients were <10 % and 20–25 %, respectively. The samples were diluted at a ratio of 1:8000 to obtain measurements within the range of the standard curve. The ELISA was performed following the manufacturer protocol. A volume of 100 µL of sample and standard was added to each well of the microplate, followed by incubation for 60 min at room temperature. The plate was washed four times, and then 100 µL of anti-IgA Detection Antibody was added to each well and incubated for an additional 60 min at room temperature. After four washes, 100 µL of HRP solution C was added to each well, and the plate was incubated for 30 min at room temperature. Subsequently, the plate was washed four times, 100 µl of TMB substrate was applied, and it was incubated in the dark at room temperature for 30 min. The reaction was stopped by adding 100 µL of stop solution per well. Absorbance was measured at 450 nm using a microplate reader (DYNEX MAGELLAN BIOSCIENCES, Chantilly, Virginia). According to the manufacturer, the antibodies in the assay specifically detect bovine IgA. No cross-reactivity has been reported with other bovine immunoglobulins or serum proteins, as assessed by immunoelectrophoresis and ELISA. In a preliminary experiment, different sample dilutions were tested and a dilution factor of 1:8,000 was selected.

### Physiological biomarkers

The cows were fixed in self-locking feeding gates to measure HR and RT. HR (in beats per minute, bpm) was recorded with a stethoscope from the left side below the shoulder. For optimal auscultation, the stethoscope head was advanced cranially beneath the anconeal musculature. To facilitate access to this anatomical region, the forelimb was gently extended by applying slight examiner pressure with the foot towards the dewclaw. Auscultation was performed for 15 s and extrapolated to 60 s. Such method, i.e., using a standardized 15 s stethoscope HR measurement, is a commonly applied approach. However, there might be a slight risk of error related to counting in veterinary field studies to capture short-term physiological responses while minimizing handling time.

RT was measured in °C using a commercially available digital thermometer (TFA Dostmann GmbH & Co KG; Wertheim). The thermometer was inserted rectally and gently pressed against the intestinal wall to ensure a reliable core body temperature.

All measurements were performed consistently by a single experienced observer throughout the study, thereby minimizing inter-observer variability and ensuring methodological consistency.

### Behavior assessment

AD was measured following the guidelines of the Welfare Quality® protocol for dairy cows (Welfare Quality®, 2009). In this regard, AD is defined as the distance between observer and cow in cm when the cow shows one of the following behavioral characteristics: stepping backward, head shaking, attempts to escape the feeding gate, or abrupt head movements. For the AD assessment, cows were restrained in self-locking feeding gates. The observer was positioned in front of the cow (2-meter distance) and gradually approached the cow with the back of the hand extended toward the muzzle. In case without cow movements, the AD was recorded as 0 cm. Each cow was tested twice within an interval of 10 s. AD was recorded in classes comprising increments of 5 cm, ranging from 0 to 200 cm.

TTI was assessed according to the method by Ebinghaus (2016), based on established temperament assessment protocols in beef cattle (Grandin, 1993; Hoppe et al., 2010). Cows were restrained in self-locking feeding gates. The observer was positioned at the left side of the cow. The observer slowly moved the back of his hand from the withers across the flank towards the udder. This procedure was carried out twice in intervals of 5 s. The cow's response was quantified using a five-point scoring system: cow stays calm, no stepping or kicking with hind legs (score 1); cow lowers the hindquarters at least during the first and second strokes or shows maximal two steps, without kicking (score 2); cow steps maximal five times, without kicking (score 3); cow steps more than five times or kicks at least once (score 4); cow is reacting violently, is panicking, or touching the cow is (barely) not possible (score 5).

### Milking robot data

Average daily milk yield (**MY**) was calculated considering the records from each milking of the cow from the period three days before BS2, implying data consistency with the genetic milkig robot trait analyses by Behren et al. (2026). Automatically recorded technical data from the milking robot reflecting characteristics of cow behavior included the no. of “kick offs” per day of the milking device (**KOD)**, the no. of incomplete milkings per day (**IMD**) and the no. of visits in the milking robot per day (**NVD**). Also, for the technical data, daily averages were calculated considering the three days before BS2. Descriptive statistics for the milking robot traits were 36.85 kg ± 8.51 for MY, 3.13 ± 0.80 for NVD, 0.13 ± 0.33 for KOD and 0.18 ± 0.38 for IMD.

### Statistical analysis

All statistical analyses were conducted using SAS OnDemand for Academics (SAS Institute Inc., Cary, NC, USA) and applying the SAS Glimmix macro (Wolfinger and O’Connell, 1993) for generalized linear mixed model (**GLMM**) analyses. IgA values were log-transformed (**logIgA**) prior to model analysis to achieve a Gaussian distribution. According to the Kolmogorov-Smirnov test, RT and HR displayed a Gaussian distribution, but dopamine, serotonin and cortisol concentrations were not-normally distributed. However, we applied the GLMM with an identity link function for the Gaussian and for the quasi-normally distributed data.

The GLMM were applied to infer: i) associations among blood biomarkers and physiological biomarkers after calving (BS1), ii) associations among blood biomarkers, physiological biomarkers and behavior traits after behavior assessments (BS2), iii) effects of blood and physiological biomarkers after calving at BS1 on blood and physiological biomarkers after behavior assessments at BS2, iv) effects of milk production and milking behavior in the AMS on stress-related biomarkers. From these generalized linear mixed model applications, also least squares means (**LSM**) for biomarkers within factor levels and significance values for respective differences of LSM, were calculated. For the multiple testing of LSM differences, Bonferroni corrections were applied in the “SAS lsmeans statement”, automatically implying adjustments of p-values.

***i) Associations among blood and physiological biomarkers after calving at BS1***

In model group 1, we investigated the relationships among blood and physiological biomarkers after calving. In consecutive runs for sub-models 1a–1f, each biomarker (cortisol, dopamine, serotonin, logIgA, HR and RT) was considered as dependent response variable. The remaining biomarkers were defined in the model as explanatory classification variables. In this regard, the classifications at BS1 were the following: 3 levels for the cortisol concentration for low: ≤ 2 ng/ml, medium: > 2–5 ng/ml and high: > 5 ng/ml; 3 levels for the dopamine concentration for low: ≤ 4 ng/ml, medium: > 4–6 ng/ml and high: > 6 ng/ml; 3 levels for the serotonin concentration for low: ≤ 25 ng/ml, medium: >25–40 ng/ml and high: > 40 ng/ml; 3 levels for the IgA concentration for low: ≤ 1.2 × 10^6^ ng/ml, medium: >1.2 × 10^6^–2.3 × 10^6^ ng/ml and high: > 2.3 × 10^6^ ng/ml; 4 levels for HR with ≤ 88 bpm, > 88–96 bpm, > 96–104 bpm and > 104 bpm; 4 levels for RT with ≤ 38.3 °C, 38.31–38.5 °C, 38.51–38.7 °C and > 38.7 °C. For the classifications, we aimed on an equal distribution of observations across levels of effects.

Model 1a was defined to infer the effects on cortisol concentrations as follows:

$$\begin{array}{c} y_{\text{ijklmnop}}=\mu +\text{Her}d_{i}+\text{Parit}y_{j}+\text{DIMPostparta}l_{k}+\text{Dopamin}e_{l}+\\ \text{Serotoni}n_{m}+\text{Ig}A_{n}+HR_{o}+RT_{p}+e_{\text{ijklmnop}}\left[1a\right] \end{array}$$

where y_ijklmnop_ = cortisol concentration at BS1; μ = overall mean; Herd_i_ = fixed effect for the herd reflecting the respective contemporary group with same feeding, management and climate conditions, etc.; Parity_j_ = fixed effect for the parity number; DIMPostpartal_k_ = fixed effect for days in milk after calving in classes (k = 0, 1–5, 6–9, 10–14 d); Dopamine_l_ = fixed effect for the dopamine concentration levels as defined above; Serotonin_m_ = fixed effect for the serotonin concentration as defined above; IgA_n_ = fixed effect for the IgA concentration as defined above; HR_o_ = fixed effect for the heart rate as defined above; RT_p_ = fixed effect for the rectal temperature as defined above; and e_ijklmnop_ = random residual effect.

The model 1b for the response variable dopamine was

$$\begin{array}{c} y_{\text{ijklmnop}}=\mu +\text{Her}d_{i}+\text{Parit}y_{j}+\text{DIMPostparta}l_{k}+\text{Cortiso}l_{l}+\text{Serotoni}n_{m}+\text{Ig}A_{n}+\\ \;\;\;\;HR_{o}+RT_{p}+e_{\text{ijklmnop}}\left[1b\right] \end{array}$$

where y_ijklmnop_ = dopamine concentration at BS1; Cortisol_l_ = fixed effect for the cortisol concentration as defined above, and all other effects as specified for model 1a.

The model 1c for the response variable serotonin was

$$\begin{array}{c} y_{\text{ijklmnop}}=\mu +\text{Her}d_{i}+\text{Parit}y_{j}+\text{DIMPostparta}l_{k}+\text{Cortiso}l_{l}+\\ \text{Dopamin}e_{m}+\text{Ig}A_{n}+HR_{o}+RT_{p}+e_{\text{ijklmnop}}\left[1c\right] \end{array}$$

where y_ijklmnop_ = serotonin concentration at BS1, and all other effects as specified for models 1a and 1b.

The model 1d for the response variable log-transformed IGA was

$$\begin{array}{c} y_{\text{ijklmnop}}=\mu +\text{Her}d_{i}+\text{Parit}y_{j}+\text{DIMPostparta}l_{k}+\text{Cortiso}l_{l}+\\ \text{Dopamin}e_{m}+\text{Serotoni}n_{n}+HR_{o}+RT_{p}+e_{\text{ijklmnop}}\left[1d\right] \end{array}$$

where y_ijklmnop_ = logIgA at BS1, and all other effects as specified for models 1a and 1b.

The model 1e for the response variable HR was

$$\begin{array}{c} y_{\text{ijklmnop}}=\mu +\text{Her}d_{i}+\text{Parit}y_{j}+\text{DIMPostparta}l_{k}+\text{Cortiso}l_{l}+\\ \text{Dopamin}e_{m}+\text{Serotoni}n_{n}+\text{Ig}A_{o}+RT_{p}+e_{\text{ijklmnop}}\left[1e\right] \end{array}$$

where y_ijklmnop_ = HR at BS1, and all other effects as specified for models 1a and 1b.

The model 1f for the response variable RT was

$$\begin{array}{c} y_{\text{ijklmnop}}=\mu +\text{Her}d_{i}+\text{Parit}y_{j}+\text{DIMPostparta}l_{k}+\text{Cortiso}l_{l}+\\ \text{Dopamin}e_{m}+\text{Serotoni}n_{n}+HR_{o}+RT_{p}+e_{\text{ijklmnop}}\left[1f\right] \end{array}$$

where y_ijklmnop_ = RT at BS1, and all other effects as specified for models 1a and 1b.

***ii) Associations among blood biomarkers, physiological biomarkers and behavior traits after behavior assessments at BS2***

In model group 2, we investigated the relationships among blood biomarkers and physiological biomarkers, as well as the effects of behavior traits on biomarkers at BS2. In analogy to models 1, in consecutive runs for sub-models 2a–2f, each biomarker (cortisol, dopamine, serotonin, logIgA, HR and RT) was considered as dependent response variable. The remaining biomarkers were considered in the model as explanatory classification variables. In this regard, the classifications at BS2 were the following: 3 levels for cortisol concentration for low: ≤ 1 ng/ml, medium: 2–3 ng/ml and high: > 3 ng/ml; 3 levels for dopamine concentration for low: ≤ 5 ng/ml, medium: > 5–12 ng/mg and high: > 12 ng/ml; 3 levels for serotonin concentration for low: ≤ 25 ng/ml, medium: > 25–35 ng/ml and high: > 35 ng/ml; 3 levels for IgA concentration for low: ≤ 2 × 10^6^ ng/ml, medium: > 2.0 × 10^6^ - 3.7 × 10^6^ ng/ml and high: > 3.7 × 10^6^ ng/ml; 4 levels for HR with ≤ 92 bpm, > 92–100 bpm, > 100–108 bpm, > 108 bmp; 4 levels for RT with ≤ 38.2 °C, 38.21–8.3 °C, 38.31–38.4 °C, > 38.4 °C; 3 levels for AD1 with ≤ 70 cm, 71–149 cm, > 150 cm; 3 levels for AD2 ≤ 50 cm, 51–139 cm, > 140 cm; 5 levels for the TTI1 scores; 5 levels for TTI2 scores. For the classifications, we aimed on an equal distribution of observations across levels of effects.

Model 2a was defined to infer the effects on cortisol concentrations and was defined as follows:

$$\begin{array}{c} y_{\text{ijklmnopqrst}}=\mu +\text{Her}d_{i}+\text{Parit}y_{j}+\text{DIMPostparta}l_{k}+\text{Dopamin}e_{l}+\\ \text{Serotoni}n_{m}+\text{Ig}A_{n}+HR_{o}+RT_{p}+\text{AD}1_{q}+\text{AD}2_{r}+\text{TTI}1_{s}+\text{TTI}2_{t}+e_{\text{ijklmnopqrst}}\left[2a\right] \end{array}$$

The model 2b for the response variable dopamine was

$$\begin{array}{c} y_{\text{ijklmnopqrst}}=\mu +\text{Her}d_{i}+\text{Parit}y_{j}+\text{DIMPostparta}l_{k}+\text{Cortiso}l_{l}+\\ \text{Serotoni}n_{m}+\text{Ig}A_{n}+HR_{o}+RT_{p}+\text{AD}1_{q}+\text{AD}2_{r}+\text{TTI}1_{s}+\text{TTI}2_{t}+e_{\text{ijklmnopqrst}}\left[2b\right] \end{array}$$

The model 2c for the response variable serotonin was

$$\begin{array}{c} y_{\text{ijklmnopqrst}}=\mu +\text{Her}d_{i}+\text{Parit}y_{j}+\text{DIMPostparta}l_{k}+\text{Cortiso}l_{l}+\\ \text{Dopamin}e_{m}+\text{Ig}A_{n}+HR_{o}+RT_{p}+\text{AD}1_{q}+\text{AD}2_{r}+\text{TTI}1_{s}+\text{TTI}2_{t}+e_{\text{ijklmnopqrst}}\left[2c\right] \end{array}$$

The model 2d for the response variable log-transformed IgA was

$$\begin{array}{c} y_{\text{ijklmnopqrst}}=\mu +\text{Her}d_{i}+\text{Parit}y_{j}+\text{DIMPostparta}l_{k}+\text{Cortiso}l_{l}+\\ \text{Dopamin}e_{m}+\text{Serotoni}n_{n}+HR_{o}+RT_{p}+\text{AD}1_{q}+\text{AD}2_{r}+\text{TTI}1_{s}+\text{TTI}2_{t}+e_{\text{ijklmnopqrs}}\left[2d\right] \end{array}$$

The model 2e for the response variable HR was

$$\begin{array}{c} y_{ijklmnopqrst}=\mu +\text{Her}d_{i}+\text{Parit}y_{j}+\text{DIMPostparta}l_{k}+\text{Cortiso}l_{l}+\\ \text{Dopamin}e_{m}+\text{Serotoni}n_{n}+\text{Ig}A_{o}+RT_{p}+\text{AD}1_{q}+\text{AD}2_{r}+\text{TTI}1_{s}+\text{TTI}2_{t}+e_{\text{ijklmnopqrst}}\left[2e\right] \end{array}$$

The model 2f for the response variable RT was

$$\begin{array}{c} y_{\text{ijklmnopqrst}}=\mu +\text{Her}d_{i}+\text{Parit}y_{j}+\text{DIMPostparta}l_{k}+\text{Cortiso}l_{l}+\\ \text{Dopamin}e_{m}+\text{Serotoni}n_{n}+\text{Ig}A_{o}+HR_{p}+\text{AD}1_{q}+\text{AD}2_{r}+\text{TTI}1_{s}+\text{TTI}2_{t}+e_{\text{ijklmnopqrst}}\left[2f\right] \end{array}$$

with the effects as defined for models 1 or introduced above for the model 2 effect classifications.

***iii) Associations between blood and physiological biomarkers at BS1 and blood and physiological biomarkers at BS2***

In model group 3, we investigated the associations between blood and physiological biomarkers at BS1 and blood and behavior biomarkers at BS2. In analogy to models 1 and 2, in consecutive runs for sub-models 3a–3f, each biomarker (cortisol, dopamine, serotonin, logIgA, HR and RT) was considered as dependent response variable. The remaining biomarkers were considered in the model as explanatory classification variables. Explanatory variables are outlined above for model 1.

Model 3a for the response variable cortisol concentrations was:

$$\begin{array}{c} y_{\text{ijklmnopq}}=\mu +\text{Her}d_{i}+\text{Parit}y_{j}+\text{DI}M_{k}+\text{Cortisol}\_\text{BS}1_{l}+\\ \text{Dopamine}\_\text{BS}1_{m}+\text{Serotonin}\_\text{BS}1_{n}+\text{IgA}\_\text{BS}1_{o}+\text{HR}\_\text{BS}1_{p}+\text{RT}\_\text{BS}1_{q}+e_{\text{ijklmnopq}}\left[3a\right] \end{array}$$

Model 3b for the response variable dopamine was

$$\begin{array}{c} y_{\text{ijklmnopq}}=\mu +\text{Her}d_{i}+\text{Parit}y_{j}+\text{DI}M_{k}+\text{Cortisol}\_\text{BS}1_{l}+\\ \text{Dopamine}\_\text{BS}1_{m}+\text{Serotonin}\_\text{BS}1_{n}+\text{IgA}\_\text{BS}1_{o}+\text{HR}\_\text{BS}1_{p}+\text{RT}\_\text{BS}1_{q}+e_{\text{ijklmnopq}}\left[3b\right] \end{array}$$

Model 3c for the response variable serotonin was

$$\begin{array}{c} y_{\text{ijklmnopq}}=\mu +\text{Her}d_{i}+\text{Parit}y_{j}+\text{DI}M_{k}+\text{Cortisol}\_\text{BS}1_{l}+\\ \text{Dopamine}\_\text{BS}1_{m}+\text{Serotonin}\_\text{BS}1_{n}+\text{IgA}\_\text{BS}1_{o}+\text{HR}\_\text{BS}1_{p}+\text{RT}\_\text{BS}1_{q}+e_{\text{ijklmnopq}}\left[3c\right] \end{array}$$

Model 3d for the response variable log-transformed IgA was

$$\begin{array}{c} y_{\text{ijklmnopq}}=\mu +\text{Her}d_{i}+\text{Parit}y_{j}+\text{DI}M_{k}+\text{Cortisol}\_\text{BS}1_{l}+\text{Dopamine}\_\text{BS}1_{m}+\\ \text{Serotonin}\_\text{BS}1_{n}+\text{IgA}\_\text{BS}1_{o}+\text{HR}\_\text{BS}1_{p}+\text{RT}\_\text{BS}1_{q}+e_{\text{ijklmnopq}}\left[3d\right] \end{array}$$

Model 3e for the response variable HR was

$$\begin{array}{c} y_{\text{ijklmnopq}}=\mu +\text{Her}d_{i}+\text{Parit}y_{j}+\text{DI}M_{k}+\text{Cortisol}\_\text{BS}1_{l}+\text{Dopamine}\_\text{BS}1_{m}+\\ \text{Serotonin}\_\text{BS}1_{n}+\text{IgA}\_\text{BS}1_{o}+\text{HR}\_\text{BS}1_{p}+\text{RT}\_\text{BS}1_{q}+e_{\text{ijklmnopq}}\left[3e\right] \end{array}$$

Model 3f for the response variable RT was

$$\begin{array}{c} y_{\text{ijklmnopq}}=\mu +\text{Her}d_{i}+\text{Parit}y_{j}+\text{DI}M_{k}+\text{Cortisol}\_\text{BS}1_{l}+\text{Dopamine}\_\text{BS}1_{m}+\\ \text{Serotonin}\_\text{BS}1_{n}+\text{IgA}\_\text{BS}1_{o}+\text{HR}\_\text{BS}1_{p}+\text{RT}\_\text{BS}1_{q}+e_{\text{ijklmnopq}}\left[3f\right] \end{array}$$

with basic explanatory variables in all models 3 as outlined above for model 1plus the following effects: Cortisol_BS1_l_ = fixed effect for the cortisol concentration levels as defined in model 1; Dopamine_BS1_m_ = fixed effect for the dopamine concentration levels as defined in model 1; Serotonin_BS1_n_ = fixed effect for the serotonin concentration levels as defined in model 1; IgA_BS1_o_ = fixed effect for the IgA concentration levels as defined in model 1; HR_BS1_p_ = fixed effect for the HR levels as defined in model 1; RT_BS1_q_ = fixed effect for the RT levels as defined in model 1

***iv) Associations between AMS milk yield, milking behavior traits and blood and physiological biomarkers at BS2***

In model group 4, we examined the associations between average milk production (MY) and milking behavior (KOD, IMD, NVD) with blood and physiological biomarkers at BS2. In consecutive runs for sub-models 4a–4d, each biomarker (cortisol, dopamine, serotonin, logIgA, HR and RT) was considered as dependent response variable. MY, KOD, IMD and NVD were considered in the model as explanatory classification variables. In this regard, the classification at BS2 were the following: 3 levels for MY in kg for low: ≤ 32.5 kg, medium: 32.5 kg–40 kg and high: > 40kg; 3 levels for the number of KOD for low: 0, medium: 0–0.5 and high: > 0.5; 3 levels for the number of IMD for low: 0, medium: 0–1 and high: >1; 3 levels for NVD for low: ≤ 2.5, medium: 2.5–3.5 and high: > 3.5. For the classifications, we aimed on an equal distribution of observations across levels of effects.

Model 4a was defined to investigate the effects of MY on blood and physiological biomarkers

$$y_{\text{ijkl}}=\mu +\text{Her}d_{i}+\text{Parit}y_{j}+\text{DI}M_{k}+MY_{l}+e_{\text{ijkl}}\left[4a\right]$$

where y_ijklmnop_ = cortisol concentration, dopamine concentration, serotonin concentration, logIgA concentration, HR and RT at BS2 in consecutive runs.

Model 4b was defined to investigate the effects of KOD on blood and physiological biomarkers

$$y_{\text{ijkl}}=\mu +\text{Her}d_{i}+\text{Parit}y_{j}+\text{DI}M_{k}+\text{KO}D_{l}+e_{\text{ijkl}}\left[4b\right]$$

where y_ijklmnop_ = cortisol concentration, dopamine concentration, serotonin concentration, logIgA concentration, HR and RT at BS2 in consecutive runs.

Model 4c was defined to investigate the effects of IMD on blood and physiological biomarkers

$$y_{\text{ijkl}}=\mu +\text{Her}d_{i}+\text{Parit}y_{j}+\text{DI}M_{k}+\text{IM}D_{l}+e_{\text{ijkl}}\left[4b\right]$$

where y_ijklmnop_ = cortisol concentration, dopamine concentration, serotonin concentration, logIgA concentration, HR and RT at BS2 in consecutive runs.

Model 4d was defined to investigate the effects of NVD on blood and physiological biomarkers

$$y_{\text{ijkl}}=\mu +\text{Her}d_{i}+\text{Parit}y_{j}+\text{DI}M_{k}+\text{NV}D_{l}+e_{\text{ijkl}}\left[4b\right]$$

where y_ijklmnop_ = cortisol concentration, dopamine concentration, serotonin concentration, logIgA concentration, HR and RT at BS2 in consecutive runs.

For all models, we used the very strict convergence criterion PCONV = 1.11022E-8, specifying parameter estimate convergence for doubly iterative estimations. Hence, in the changes after two consecutive iterations, alterations for solutions of effects as well as for variance components, were considered simultaneously.

## Results

### Descriptive statistics of blood and physiological biomarkers after calving and behavioral assessment

Descriptive statistics for blood and physiological biomarkers at BS1 and BS2 are presented in Table 1. The mean values of the individual biomarkers indicated only minor differences between BS1 and BS2.

**Table 1:** Descriptive statistics for biomarkers of cows at blood sampling 1 (BS1) and blood sampling 2 (BS2), and respective reference values from our previous pilot study^2^ considering cows under acute stress in a claw care crush.

Biomarkers^1^	BS1			BS2			Pilot study^2^		
	Mean	± SD	(Min-Max)	Mean	± SD	(Min-Max)	Mean	± SD	(Min-Max)
Cortisol (ng/ml)	4.74	4.67	(0.30–28.76)	4.94	4.47	(0.30–29.03)	28.31	13.03	(6.93–40.00)
Dopamin (ng/ml)	6.67	8.1	(0.89–112.98)	13.68	16.13	(0.19–112.10)	12.07	11.61	(2.01–18.98)
Serotonin (ng/ml)	43.65	54.35	(3.21–665.50)	33.44	16.64	(2.68–183.45)	20.51	21.16	(9.89–79.25)
IgA (ng/ml)	2.86×10^6^	7×10^6^	(1.4×10^5^–1.12×10^8^)	3.66×10^6^	2.64×10^6^	(4.12×10^5^–1.39×10^7^)	1.44×10^6^	6.13×10^5^	(9.27×10^5^–1.67×10^6^)
HR	101.1	15.21	(72–164)	103.15	15.03	(68–160)	-	-	-
RT	38.62	0.4	(37.6–40.5)	38.38	0.2	(37.7–39.4)	-	-	-

^1^IgA = immunoglobulin A, HR = heart rate (beats/min), RT = rectal temperature (°C)

### Associations between blood and physiological biomarkers after calving at BS1

F-Statistics (Type III test of fixed effects), LSM and significances of pairwise comparisons from model 1 are presented in Table 2. The 13 cows with blood samplings directly at the calving date had a significantly higher (*P* < 0.001) LSM for cortisol (6.74 ± 0.51 ng/ml) than the 14 cows sampled 14 d postpartum (4.37 ± 0.26 ng/ml). The dopamine concentration (*P* < 0.001) significantly influenced the serotonin concentration, and vice versa (*P* < 0.001). Accordingly, the serotonin concentration was significantly lower in cows with low dopamine concentrations compared to cows with high dopamine concentrations (27.15 ± 4.31 ng/ml and 60.32 ± 4.05 ng/ml, respectively) (*P* < 0.001).

**Table 2:** Least square means (LSM) with corresponding SE from model 1 for blood and physiological biomarkers at blood sampling 1 (BS1) after calving.

Blood and physiological biomarkers^2^								
Fixed effect^1^	Effect class	N	Cortisol	Dopamine	Serotonin	logIgA	HR	RT
Herd	1	52	5.25 ± 0.45^a^	12.02 ± 1.34^a^	18.68 ± 6.21^b^	13.65 ± 0.13^d^	95.12 ± 2.37^c^	38.63 ± 0.07^a^
	2	93	5.11 ± 0.34^a^	4.86 ± 1.02^b^	45.21 ± 5.11^a^	14.78 ± 0.10^a^	99.24 ± 1.81^bc^	38.54 ± 0.05^b^
	3	73	5.08 ± 0.35^a^	5.97 ± 1.04^b^	53.15 ± 5.16^a^	14.74 ± 0.10^ba^	105.38 ± 1.79^a^	38.60 ± 0.05^ab^
	4	86	4.75 ± 0.34^a^	6.47 ± 1.02^b^	25.11 ± 4.91^b^	14.48 ± 0.10^b^	99.08 ± 1.83^bc^	38.62 ± 0.05^ab^
	5	93	5.22 ± 0.31^a^	5.12 ± 0.92^b^	50.86 ± 4.65^a^	14.17 ± 0.09^c^	101.22 ± 1.63^ab^	38.70 ± 0.05^a^
	*P*-value^3^	*-*	0.8302	0.0003	<.0001	<.0001	0.0089	0.2288
Parity	1	186	4.89 ± 0.26^a^	8.75 ± 0.76^a^	40.76 ± 3.84^a^	14.47 ± 0.08^a^	109.00 ± 1.22^a^	38.64 ± 0.04^ab^
	2	113	5.27 ± 0.28^a^	6.05 ± 0.84^b^	38.09 ± 4.21^a^	14.33 ± 0.08^a^	97.72 ± 1.46^b^	38.54 ± 0.04^b^
	3	98	5.09 ± 0.30^a^	5.86 ± 0.91^b^	36.96 ± 4.55^a^	14.29 ± 0.09^a^	93.30 ± 1.56^c^	38.67 ± 0.05^a^
	*P*-value	-	0.5872	0.0194	0.8043	0.2533	<.0001	0.0482
DIM Postpartal (days)	0	38	6.74 ± 0.51^a^	6.55 ± 1.51^a^	35.29 ± 7.64^a^	14.53 ± 1.51^a^	108.64 ± 2.60^a^	38.62 ± 0.08^a^
	1–5	124	4.92 ± 0.25^b^	7.96 ± 0.76^a^	41.89 ± 3.77^a^	14.46 ± 0.76^a^	99.32 ± 1.34^b^	38.65 ± 0.09^a^
	6–9	106	4.30 ± 0.28^b^	5.77 ± 0.84^a^	37.07 ± 4.21^a^	14.26 ± 0.84^a^	95.06 ± 1.45^c^	38.64 ± 0.04^a^
	10–14	128	4.37 ± 0.26^b^	7.28 ± 0.78^a^	40.16 ± 3.98^a^	14.21 ± 0.78^a^	97.01 ± 1.40^bc^	38.56 ± 0.04^a^
	*P*-value	-	0.0003	0.2376	0.7588	0.0649	0.0001	0.3073
Cortisol	Low (≤2)	119	-	6.62 ± 2.52^a^	46.86 ± 14.55^a^	13.51 ± 0.29^c^	106.65 ± 5.09^a^	38.70 ± 0.14^a^
(ng/ml)	Medium (3–5)	117	-	7.85 ± 1.87^a^	37.27 ± 9.46^a^	14.41 ± 0.19^b^	97.14 ± 3.30^a^	38.64 ± 0.09^a^
	High (>5)	117	-	6.20 ± 2.52^a^	31.67 ± 12.69^a^	15.17 ± 0.25^a^	96.23 ± 4.45^a^	38.51 ± 0.13^a^
	*P*-value	-	-	0.8394	0.8382	0.0022	0.3914	0.7019
Dopamine	Low (≤4)	96	4.74 ± 0.30^a^	-	27.15 ± 4.31^b^	14.44 ± 0.09^a^	98.35 ± 1.58^a^	38.59 ± 0.05^a^
(ng/ml)	Medium (5–6)	137	5.39 ± 0.24^a^	-	28.33 ± 3.63^b^	14.32 ± 0.07^a^	101.43 ± 1.29^a^	38.64 ± 0.04^a^
	High (>6)	113	5.12 ± 0.28^a^	-	60.32 ± 4.05^a^	14.33 ± 0.08^a^	100.25 ± 1.47^a^	38.62 ± 0.04^a^
	*P*-value	-	0.1915	-	<.0001	0.4712	0.2557	0.6061
Serotonin	Low (≤25)	134	5.11 ± 0.26^a^	4.14 ± 0.77^b^	-	14.43 ± 0.08^a^	101.34 ± 1.40^a^	38.58 ± 0.04^b^
(ng/ml)	Medium (26–40)	98	5.24 ± 0.29^a^	5.72 ± 0.87^b^	-	14.32 ± 0.09^a^	98.77 ± 1.55^a^	38.70 ± 0.04^a^
	High (>40)	117	4.89 ± 0.31^a^	10.81 ± 0.87^a^	-	14.31 ± 0.09^a^	99.92 ± 1.62^a^	38.58 ± 0.06^b^
	*P*-value	-	0.666	<.0001	-	0.6416	0.4432	0.0460
IgA	Low (≤1.2 × 10^6^)	107	5.19 ± 0.29^a^	7.65 ± 0.86^a^	36.20 ± 4.34^a^	-	100.44 ± 1.52^a^	38.63 ± 0.04^a^
(ng/ml)	Medium (>1.2 × 10^6^–2.3 × 10^6^)	121	5.01 ± 0.26^a^	5.72 ± 0.78^a^	40.24 ± 3.95^a^	-	99.10 ± 1.39^a^	38.61 ± 0.04^a^
	High (>2.3 × 10^6^)	122	5.05 ± 0.27^a^	7.30 ± 0.82^a^	39.36 ± 4.08^a^	-	100.49 ± 1.44^a^	38.61 ± 0.04^a^
	*P*-value	-	0.8863	0.1430	0.7566	-	0.6678	0.9591
HR	≤88	89	4.85 ± 0.31^a^	7.76 ± 0.93^a^	38.87 ± 4.70^a^	14.42 ± 0.09^a^	-	38.59 ± 0.05^a^
(bpm)	89–96	92	5.03 ± 0.32^a^	7.62 ± 0.94^a^	39.62 ± 4.69^ab^	14.37 ± 0.09^a^	-	38.60 ± 0.05^a^
	97–104	93	4.95 ± 0.33^a^	6.25 ± 0.97^a^	33.98 ± 4.91^b^	14.30 ± 0.10^a^	-	38.60 ± 0.05
	>104	123	5.51 ± 0.29^a^	5.93 ± 0.86^a^	41.93 ± 4.38^ab^	14.37 ± 0.09^a^	-	38.68 ± 0.04^a^
	*P*-value	-	0.3942	0.4113	0.6052	0.8447	-	0.4784
RT	≤38.3	89	4.97 ± 0.32^ab^	6.93 ± 0.95^a^	46.50 ± 4.71^a^	14.26 ± 0.10^b^	97.81 ± 1.68^a^	-
(°C)	38.31–38.5	111	5.08 ± 0.28^ab^	7.58 ± 0.84^a^	37.70 ± 4.21^a^	14.52 ± 0.08^a^	100.76 ± 1.47^a^	-
	38.51–38.7	86	4.68 ± 0.32^b^	6.52 ± 0.95^a^	33.71 ± 4.81^a^	14.35 ± 0.10^ab^	99.77 ± 1.69^a^	-
	>38.7	111	5.60 ± 0.29^b^	6.53 ± 0.85^a^	36.50 ± 4.30^a^	14.33 ± 0.09^ab^	101.69 ± 1.49^a^	-
	*P*-value	-	0.1401	0.7597	0.2082	0.1635	0.3237	-

^1^DIMPostpartal = days between calving and blood sampling, IgA = immunoglobulin A, HR = heart rate (beats/min), RT = rectal temperature (°C)

^2^logIgA = log-transformed immunoglobulin A, HR = heart rate (beats/min), RT = rectal temperature (°C)

^3^*P*-value = significance of fixed effects by F-statistics; Type III test of fixed effects

^a-c^significances for pairwise comparisons are indicated with different letters (*P* < 0.05)

Immunoglobulin A was significantly influenced by the cortisol concentration (*P* = 0.002). The LSM for logIgA was significantly lower in cows with low cortisol concentrations (13.51 ± 0.29 ng/ml) compared to cows with higher cortisol concentrations (15.17 ± 0.25 ng/ml) (*P* < 0.001).

Spearman correlation coefficients among biomarkers after calving at BS1 support the LSM results and are presented in the supplementary material S2, Table S2_1.

### Associations among blood biomarkers, physiological biomarkers and behavior traits after behavior assessments at BS2

F-Statistics, LSM and significances of pairwise comparisons from model 2 are presented in Table 3. Blood biomarkers and physiological biomarkers had no significant effects (*P* > 0.05) on the cortisol concentrations at BS2. The farm (*P* < 0.001) and serotonin concentration had a significant effect (*P* < 0.001) on the dopamine concentration. The LSM for the dopamine concentration was 7.15 ng/ml, 8.07 ng/ml and 20.65 ng/ml in cows with serotonin concentrations of < 25 ng/ml, 26–35 ng/ml and > 35 ng/ml, respectively. The LSM difference between the low and high groups was significant (*P* < 0.05), but non-significant (*P* > 0.05) between intermediate and high, and between intermediate and low cow groups.

**Table 3:** Least square (LSM) means with corresponding SE from model 2 for blood and physiological biomarkers at blood sampling 2 (BS2) after behavior tests.

Blood and physiological biomarkers^2^								
Fixed effect^1^	Effect class	N	Cortisol	Dopamine	Serotonin	logIgA	HR	RT
Herd	1	37	4.21 ± 0.70^a^	18.40 ± 3.94^a^	24.09 ± 4.09^bc^	14.73 ± 0.18^b^	104.02 ± 3.89^b^	38.28 ± 0.06^a^
	2	75	4.81 ± 0.67^a^	14.56 ± 3.69^a^	29.31 ± 3.87^b^	15.20 ± 0.17^a^	104.31 ± 3.65^b^	38.32 ± 0.05^a^
	3	60	4.42 ± 0.72^a^	14.16 ± 3.89^ab^	22.37 ± 4.05^c^	14.94 ± 0.18^ab^	112.73 ± 1.79^a^	38.35 ± 0.06^a^
	4	59	4.17 ± 0.69^a^	8.77 ± 3.83^bc^	29.92 ± 4.03^b^	15.05 ± 0.18^a^	110.60 ± 3.76^ab^	38.33 ± 0.06^a^
	5	85	4.80 ± 0.67^a^	3.90 ± 3.65^c^	39.10 ± 3.86^a^	14.96 ± 0.17^ab^	109.81 ± 3.63^ab^	38.34 ± 0.05^a^
	*P*-value^3^	-	0.6118	<.0001	<.0001	0.0321	0.0082	0.7275
Parity	1	129	4.39 ± 0.63^a^	12.16 ± 3.46^a^	29.06 ± 3.62^a^	14.93 ± 0.16^a^	108.71 ± 3.40^a^	38.35 ± 0.05^a^
	2	103	4.89 ± 0.62^a^	10.17 ± 3.43^a^	27.50 ± 3.59^a^	14.97 ± 0.16^a^	106.60 ± 3.39^a^	38.32 ± 0.05^a^
	3	84	4.17 ± 0.65^a^	13.55 ± 3.62^a^	30.31 ± 3.78^a^	15.02 ± 0.17^a^	109.57 ± 3.54^a^	38.30 ± 0.05^a^
	*P*-value	-	0.1906	0.3212	0.4977	0.6752	0.3798	0.3060
DIM	70–120	69	4.50 ± 0.68^a^	15.86 ± 3.76^a^	27.30 ± 3.92^a^	15.14 ± 0.17^a^	110.01 ± 3.70^a^	38.31 ± 0.05^a^
	121–180	82	4.76 ± 0.65^a^	10.20 ± 3.61^b^	30.55 ± 3.78^a^	14.94 ± 0.17^ab^	106.87 ± 3.57^a^	38.31 ± 0.05^a^
	181–239	73	4.34 ± 0.63^a^	8.83 ± 3.48^b^	28.37 ± 3.64^a^	14.90 ± 0.16^b^	108.73 ± 3.41^a^	38.35 ± 0.05^a^
	≥240	92	4.32 ± 0.64^a^	12.93 ± 3.55^ab^	29.62 ± 3.71^a^	14.92 ± 0.16^ab^	107.56 ± 3.49^a^	38.32 ± 0.05^a^
	*P*-value	-	0.7308	0.0363	0.6272	0.1670	0.6088	0.5118
Cortisol	Low (≤ 1)	37	-	15.03 ± 4.41^a^	29.14 ± 4.63^a^	15.00 ± 0.21^a^	106.14 ± 4.37^a^	38.24 ± 0.06^b^
(ng/ml)	Medium (2–3)	106	-	9.88 ± 3.45^a^	30.39 ± 3.60^a^	15.02 ± 0.16^a^	107.25 ± 3.38^a^	38.34 ± 0.05^a^
	High (>3)	173	-	10.97 ± 3.70^a^	27.35 ± 3.86^a^	15.17 ± 0.17^a^	111.49 ± 3.61^a^	38.38 ± 0.05^a^
	*P*-value	-	-	0.3013	0.5406	0.6980	0.2820	0.0808
Dopamine	Low (≤ 5)	87	4.40 ± 0.64^a^	-	22.73 ± 3.66^b^	15.05 ± 0.16^a^	108.28 ± 3.48^a^	38.35 ± 0.05^a^
(ng/ml)	Medium (6–12)	112	4.44 ± 0.63^a^	- -	25.07 ± 3.67^b^	14.96 ± 0.16^a^	109.17 ± 3.47^a^	38.29 ± 0.05^a^
	High (>12)	109	4.61 ± 0.65^a^	-	39.08 ± 3.70^a^	14.91 ± 0.17^a^	107.44 ± 3.50^a^	38.32 ± 0.05^a^
	*P*-value	-	0.8823	-	<.0001	0.4939	0.7397	0.2295
Serotonin	Low (≤ 25)	87	4.64 ± 0.62^a^	7.15 ± 3.45^b^	-	14.93 ± 0.16^a^	106.85 ± 3.40^a^	38.33 ± 0.05^a^
(ng/ml)	Medium (26–35)	123	4.48 ± 0.63^a^	8.07 ± 3.48^b^	-	14.90 ± 0.16^a^	108.80 ± 3.42^a^	38.34 ± 0.04^a^
	High (>35)	106	4.32 ± 0.66^a^	20.65 ± 3.58^a^	-	15.00 ± 0.17^a^	109.24 ± 3.57^a^	38.30 ± 0.05^a^
	*P*-value	-	0.7811	<.0001	-	0.7907	0.5648	0.3760
IgA	Low (≤ 2 × 10^6^)	93	4.78 ± 0.64^a^	13.30 ± 3.32^a^	25.67 ± 3.78^b^	-	107.86 ± 3.57^a^	38.31 ± 0.05^a^
(ng/ml)	Medium (>2 × 10^6^–3.7 × 10^6^)	111	4.23 ± 0.63^a^	12.01 ± 3.12^a^	30.19 ± 3.56^a^	-	107.74 ± 3.36^a^	38.31 ± 0.05^a^
	High (3.7 × 10^6^)	112	4.44 ± 0.65^a^	10.56 ± 3.19^a^	31.02 ± 3.62^a^	-	109.28 ± 3.38^a^	38.35 ± 0.05^a^
	*P*-value	-	0.3876	0.4633	0.0507	-	0.7109	0.3711
HR	≤ 92	92	4.63 ± 0.66^a^	10.74 ± 3.67^ab^	28.95 ± 3.83a	15.03 ± 0.17^a^	-	38.34 ± 0.05^a^
(bpm)	93–100	69	4.11 ± 0.66^a^	12.45 ± 3.66^ab^	29.92 ± 3.83^a^	14.85 ± 0.17^a^	-	38.36 ± 0.05^a^
	101–108	63	4.49 ± 0.67^a^	9.72 ± 3.72^b^	27.61 ± 3.88^a^	14.98 ± 0.17^a^	-	38.32 ± 0.05^ab^
	>108	92	4.70 ± 0.61^a^	14.93 ± 3.41^a^	29.36 ± 3.56^a^	15.05 ± 0.16^a^	-	38.26 ± 0.05^b^
	*P*-value	-	0.5666	0.1840	0.8632	0.3049	-	0.0281
RT	≤ 38.2	75	3.94 ± 0.63^b^	12.83 ± 3.46^a^	29.16 ± 3.61^a^	14.88 ± 0.16^a^	109.83 ± 3.37^a^	-
(°C)	38.21–38.3	77	4.66 ± 0.66^ab^	11.30 ± 3.68^a^	29.71 ± 3.85^a^	15.00 ± 0.17^a^	110.46 ± 3.60^a^	-
	38.31–38.4	66	4.43 ± 0.67^ab^	10.62 ± 3.71^a^	26.74 ± 3.88^a^	14.99 ± 0.17^a^	105.92 ± 3.66^a^	-
	>38.4	98	4.90 ± 0.64^a^	13.09 ± 3.56^a^	30.22 ± 3.73^a^	15.03 ± 0.17^a^	106.97 ± 3.53^a^	-
	*P*-value	-	0.1584	0.7031	0.5817	0.5948	0.1893	-
AD1	≤ 70	106	4.85 ± 0.66^a^	11.55 ± 3.67^a^	27.12 ± 3.82^a^	15.09 ± 0.17^a^	108.77 ± 3.61^a^	38.32 ± 0.05^a^
(cm)	71–149	114	4.44 ± 0.63^a^	11.16 ± 3.52^a^	27.80 ± 3.68^a^	14.91 ± 0.16^a^	108.38 ± 3.46^a^	38.32 ± 0.05^a^
	>150	92	4.16 ± 0.64^a^	13.15 ± 3.55^a^	31.96 ± 3.72^a^	14.92 ± 0.16^a^	107.74 ± 3.48^a^	38.33 ± 0.05^a^
	*P*-value	-	0.4156	0.6933	0.1853	0.2402	0.9351	0.8578
AD2	≤ 50	110	4.36 ± 0.65^a^	11.93 ± 3.60^a^	30.11 ± 3.76^a^	14.91 ± 0.17^a^	105.68 ± 3.55^b^	38.33 ± 0.05^a^
(cm)	51–139	95	4.19 ± 0.65^a^	10.13 ± 3.58^a^	28.12 ± 3.73^a^	15.05 ± 0.17^a^	110.53 ± 3.50^a^	38.33 ± 0.05^a^
	> 140	107	4.90 ± 0.64^a^	13.81 ± 3.54^a^	28.65 ± 3.71^a^	14.96 ± 0.16^a^	108.67 ± 3.48^ab^	38.31 ± 0.05^a^
	*P*-value	-	0.2525	0.2862	0.7290	0.4039	0.1226	0.7815
TTI1	1	136	3.97 ± 0.67^a^	13.38 ± 3.70^a^	27.34 ± 3.86^a^	14.89 ± 0.17^a^	104.14 ± 3.65^b^	38.29 ± 0.05^a^
	2	76	4.46 ± 0.68^a^	10.37 ± 3.79^a^	27.40 ± 3.95^a^	15.05 ± 0.18^a^	107.83 ± 3.72^b^	38.31 ± 0.06^a^
	3	59	4.81 ± 0.68^a^	10.48 ± 3.77^a^	29.30 ± 3.93^a^	15.07 ± 0.17^a^	106.48 ± 3.72^b^	38.31 ± 0.06^a^
	4	45	4.69 ± 0.67^a^	13.61 ± 3.68^a^	31.79 ± 3.86^a^	14.89 ± 0.17^a^	114.73 ± 3.59^a^	38.38 ± 0.05^a^
	5	0	-	-	-	-	-	-
	*P*-value	-	0.7308	0.0363	0.6272	0.1670	0.6088	0.5118
TTI2	1	190	4.73 ± 0.26^a^	14.75 ± 1.65^a^	34.21 ± 1.73^a^	14.83 ± 0.08^a^	102.01 ± 1.63^a^	38.38 ± 0.02^a^
	2	67	4.52 ± 0.36^a^	15.27 ± 2.06^a^	30.04 ± 2.17^a^	15.05 ± 0.10^a^	104.98 ± 2.05^a^	38.35± 0.03^a^
	3	40	5.22 ± 0.47^a^	20.76 ± 2.69^a^	28.33 ± 2.88^a^	14.95 ± 0.13^a^	107.38 ± 2.72^a^	38.36 ± 0.04^a^
	4	18	3.80 ± 0.71^a^	8.15 ± 3.94^a^	30.03 ± 4.13^a^	14.70 ± 0.18^a^	105.60 ± 3.90^a^	38.33 ± 0.21^a^
	5	1	4.14 ± 2.79^a^	0.86 ± 15.28^a^	22.18 ± 16.00^a^	15.35 ± 0.71^a^	121.52 ± 15.10^a^	38.20 ± 0.21^a^
	*P*-value	-	0.5187	0.0509	0.2626	0.1825	0.2770	0.8368

^1^DIM= days in milk, IgA = immunoglobulin A, HR = heart rate (beats/min), RT = rectal temperature (°C), AD1 = first observation of avoidance distance; ^2^AD2 = second observation of avoidance distance, TTI1 = first observation of tolerance to tactile interaction, TTI2 = second observation of tolerance to tactile interaction

^2^logIgA = log-transformed immunoglobulin A, HR = heart rate (beats/min), RT = rectal temperature (°C)

^3^*P*-value = significance of fixed effects by F-statistics; Type III test of fixed effects

^a-c^significances for pairwise comparisons are indicated with different letters (*P* < 0.05)

Vice versa, the serotonin concentration was significantly influenced by the dopamine concentration (*P* < 0.001). Cows with low dopamine concentrations had significantly lower serotonin concentrations (LSM = 22.73 ±3.66 ng/ml) compared to cows with high dopamine concentrations in serum (LSM = 39.08 ± 3.70 ng/ml) (*P* < 0.001).

The fixed effect TTI1 (*P* = 0.012) significantly affected HR. Cows with TTI1 score 1 showed a significantly lower LSM for HR (104.14 ± 3.64 bpm) than cows with the highest TTI1 score 5 (114.73 ± 3.59 bpm) (*P* = 0.001). In addition, HR (*P* = 0.028) had a significant influence on RT. Cows with a low HR had a significantly higher LSM for RT (38.34 ± 0.05 °C) than cows with a high HR (38.26 ± 0.05 °C) (*P* < 0.05).

Spearman correlation coefficients among biomarkers after behavior tests at BS2 support the LSM results and are presented in the supplementary material S2, Table S2_2.

### Associations between blood and physiological biomarkers at BS1 and blood and physiological biomarkers at BS2

**Fig. 3:**
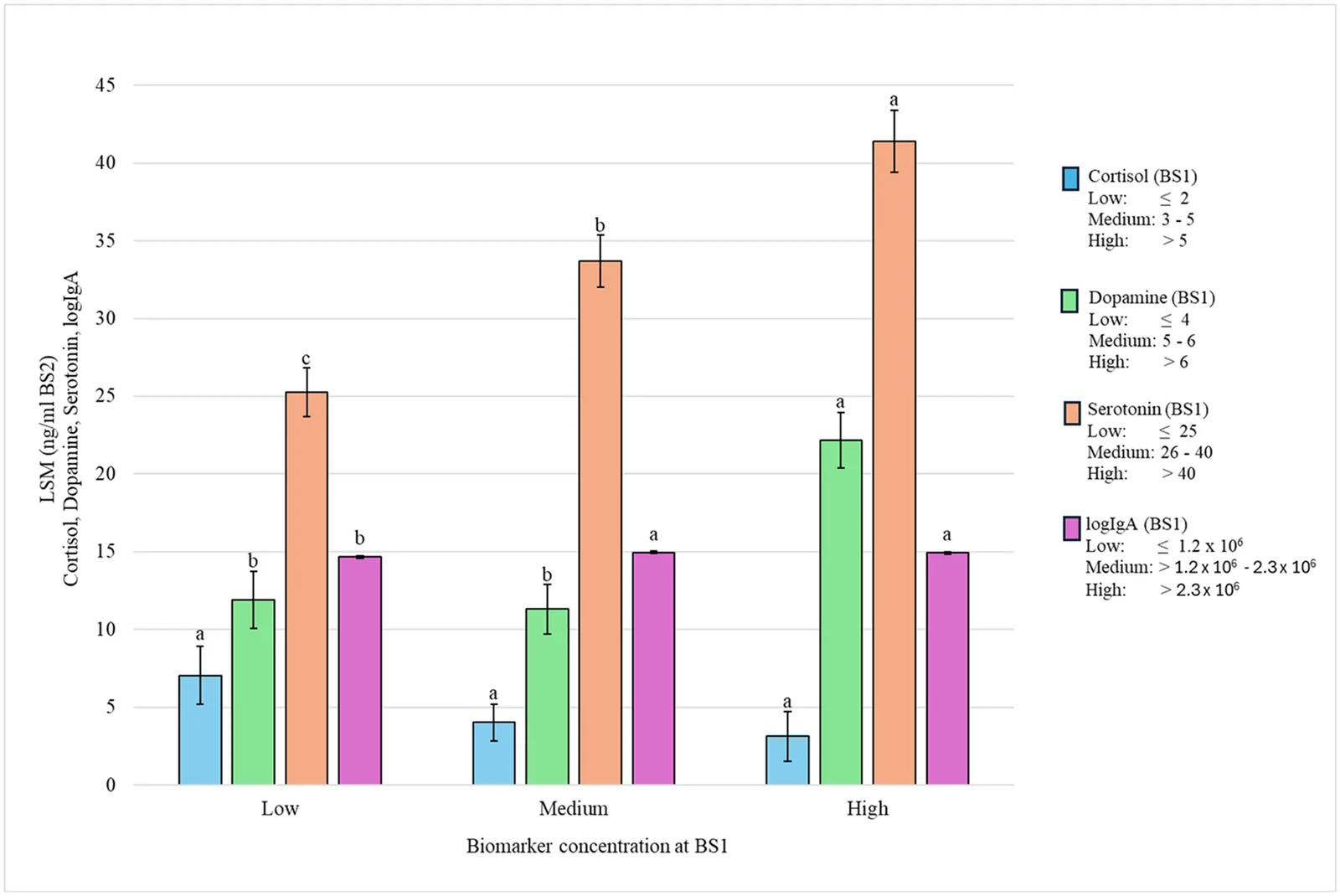
Least squares means (LSM) for cortisol, dopamine, serotonin, and logIgA at blood sampling 2 (BS2; after behavior assessment) in dependency of concentrations at blood sampling 1 (BS1; after calving) and. Different letters within classes of biomarkers indicate significant differences (*P* < 0.05).

F-Statistics, LSM and significances of pairwise comparisons from model 3 are presented in Table 4. The LSM for cortisol at BS2 with respect to the cortisol classes at BS1were not significantly different (*P* > 0.05). The interval between both measurement dates was quite large (average 181 days), but some significant associations regarding BS1 classifications were observed for serotonin, dopamine and logIgA. Fig. 3 illustrates such association pattern. Low LSM for dopamine concentrations at BS1 were associated with lower values at BS2 (11.88 ± 1.83 ng/ml), while high dopamine concentrations at BS1 resulted in significantly higher LSM (*P* < 0.05) for dopamine concentrations at BS2 (22.16 ± 1.78 ng/ml) (Fig. 3; Table 5). Similarly, cows with low serotonin concentrations (≤ 25 ng/ml) at BS1 also had significantly lower LSM (*P* < 0.05) for serotonin concentrations at BS2 (25.26 ± 1.56 ng/ml). In analogy, high serotonin concentrations at BS1 (> 40 ng/ml) were associated with significantly higher LSM (*P* < 0.05) for serotonin concentrations at BS2 (41.40 ± 1.96 ng/ml) (Fig. 3; Table 5).

**Table 4:** Least square means (LSM) with corresponding SE from model 3 for blood and physiological biomarkers between blood sampling 1 (BS1) and blood sampling 2 (BS2).

Blood and physiological biomarkers^2^								
Fixed effect^1^	Effect class	N	Cortisol	Dopamine	Serotonin	logIgA	HR	RT
Herd	1	37	3.49 ± 0.91^b^	18.91 ± 3.28^a^	33.66 ± 2.93^cb^	14.68 ± 0.14^b^	101.02 ± 2.93^ab^	38.31 ± 0.04^a^
	2	75	4.84 ± 0.62^ab^	16.30 ± 2.07^ab^	30.92 ± 1.98^c^	15.06 ± 0.10^a^	98.95 ± 1.98^b^	38.35 ± 0.03^a^
	3	60	5.90 ± 0.63^a^	14.11 ± 2.12^ab^	23.62 ± 2.03^d^	14.73 ± 0.10^b^	106.48 ± 2.03^a^	38.40 ± 0.03^a^
	4	59	4.72 ± 0.66^ab^	15.12 ± 2.22^ab^	38.48 ± 2.12^ab^	14.85 ± 0.10^ab^	103.55 ± 2.11^ab^	38.38 ± 0.03^a^
	5	85	4.74 ± 0.55^ab^	11.14 ± 1.86^b^	40.56 ± 1.78^a^	14.87 ± 0.09^ab^	103.65 ± 1.78^ab^	38.37 ± 0.03^a^
	*P*-value	-	0.3405	0.1992	<.0001	0.0869	0.0763	0.421
Parity	1	129	4.98 ± 0.48^a^	13.10 ± 1.63^a^	32.23 ± 1.54^a^	14.77 ± 0.07^a^	100.29 ± 1.54^b^	38.40 ± 0.02^a^
	2	103	4.88 ± 0.50^a^	14.18 ± 1.71^a^	32.27 ± 1.62^a^	14.87 ± 0.08^a^	102.33 ± 1.62^ab^	38.36 ± 0.02^ab^
	3	84	4.36 ± 0.55^a^	18.08 ± 1.89^a^	35.84 ± 1.76^a^	14.89 ± 0.09^a^	105.56 ± 1.76^a^	38.32 ± 0.03^b^
	*P*-value	-	0.6721	0.1228	0.2637	0.5458	0.0894	0.0639
DIM	70–120	69	4.16 ± 0.66^a^	20.45 ± 2.33^a^	37.26 ± 2.14^a^	14.92 ± 0.10^a^	103.78 ± 2.13^a^	38.31 ± 0.03^b^
	121–180	82	5.41 ± 0.56^a^	15.33 ± 1.91^ab^	36.70 ± 1.79^a^	14.85 ± 0.09^a^	99.81 ± 1.79^a^	38.37 ± 0.03^ab^
	181–239	73	5.07 ± 0.55^a^	12.25 ± 1.85^b^	31.42 ± 1.77^b^	14.80 ± 0.09^a^	102.68 ± 1.77^a^	38.40 ± 0.03^a^
	≥240	92	4.31 ± 0.53^a^	12.44 ± 1.78^b^	28.41 ± 1.70^b^	14.79 ± 0.08^a^	104.65 ± 1.70^a^	38.37 ± 0.02^ab^
	*P*-value	-	0.3071	0.0299	0.0007	0.7582	0.2231	0.1387
Cortisol_BS1	Low (≤2)	88	7.05 ± 1.86^a^	25.72 ± 6.24^a^	69.86 ± 5.99^a^	15.17 ± 0.29^a^	105.90 ± 5.98^a^	38.34 ± 0.09^a^
(ng/ml)	Medium (3–5)	98	4.03 ± 1.19^a^	13.69 ± 4.00^a^	18.10 ± 3.82^b^	14.59 ± 0.19^a^	100.06 ± 3.81^a^	38.37 ± 0.06^a^
	High (>5)	95	3.14 ± 1.61^a^	5.95 ± 5.39	12.39 ± 5.12^b^	14.77 ± 0.25^a^	102.23 ± 5.16^a^	38.38 ± 0.07^a^
	*P*-value	-	0.4598	0.1998	0.3861	0.3031	0.7420	0.9687
Dopamine_BS1	Low (≤4)	82	4.32 ± 0.54^a^	11.88 ± 1.83^b^	30.55 ± 1.74^b^	14.84 ± 0.08^a^	104.21 ± 1.74^ab^	38.38 ± 0.03^a^
(ng/ml)	Medium (5–6)	110	4.56 ± 0.45^a^	11.32 ± 1.60^b^	29.38 ± 1.46^b^	14.84 ± 0.07^a^	104.27 ± 1.46^a^	38.35 ± 0.02^a^
	High (>6)	93	5.33 ± 0.53^a^	22.16 ± 1.78^a^	40.42 ± 1.70^a^	14.84 ± 0.08^a^	99.71 ± 1.70^b^	38.35 ± 0.02^a^
	*P*-value	-	0.3979	<.0001	<.0001	0.9996	0.1019	0.5184
Serotonin_BS1 (ng/ml)	Low (≤25)	4	4.84 ± 0.48^a^	12.30 ± 1.64^b^	25.26 ± 1.56^c^	14.88 ± 0.08^a^	105.78 ± 1.56^a^	38.37 ± 0.02^a^
	Medium (26–40)	85	5.23 ± 0.52^a^	14.69 ± 1.79^ab^	33.69 ± 1.68^b^	14.82 ± 0.08^a^	101.19 ± 1.68^a^	38.36 ± 0.02^a^
	High (>40)	95	4.15 ± 0.61^a^	13.37 ± 2.09^a^	41.40 ± 1.97^a^	14.82 ± 0.10^a^	101.22 ± 1.97^a^	38.37 ± 0.03^a^
	*P*-value	-	0.3492	0.1164	<.0001	0.8710	0.1265	0.9291
IgA_BS1 (ng/ml)	Low (≤1.2 × 10^6^)	90	5.07 ± 0.52^a^	13.96 ± .78^a^	33.83 ± 1.67^ab^	14.66 ± 0.08^b^	100.52 ± 1.67^a^	38.37 ± 0.02^a^
	Medium (>1.2 × 10–2.3 × 10^6^)	98	4.42 ± 0.48^a^	14.85 ± 1.63^a^	30.50 ± 1.56^b^	14.95 ± 0.08^a^	104.40 ± 1.55^a^	38.36 ± 0.02^a^
	High (>2.3 × 10^6^)	100	4.72 ± 0.49^a^	16.55 ± 1.68^a^	36.02 ± 1.59^a^	14.92 ± 0.08^a^	103.28 ± 1.59^a^	38.35 ± 0.02^a^
	*P*-value	-	0.6406	0.5582	0.0333	0.9996	0.1019	0.5184
HR_BS1	≤88	74	4.43 ± 0.56^a^	12.54 ± 1.89^a^	34.88 ± 1.81^a^	14.81 ± 0.09^a^	94.98 ± 1.79^c^	38.36 ± 0.03^a^
(bpm)	89–96	75	5.01 ± 0.57^a^	14.69 ± 1.95^a^	33.82 ± 1.67^a^	14.83 ± 0.09^a^	100.54 ± 1.85^b^	38.38 ± 0.03^a^
	97–104	76	5.39 ± 0.58^a^	16.35 ± 1.96^a^	32.13 ± 1.96^a^	14.83 ± 0.09^a^	105.43 ± 1.87^ab^	38.36 ± 0.03^a^
	>104	88	4.13 ± 0.56^a^	16.89 ± 1.94^a^	32.97 ± 1.78^a^	14.90 ± 0.09^a^	109.96 ± 1.79^a^	38.35 ± 0.03^a^
	*P*-value	-	0.3253	0.4289	0.5364	0.8800	<.0001	0.8228
RT_BS1	≤38.3	75	4.46 ± 0.56^a^	16.09 ± 1.90^a^	32.12 ± 1.79^a^	14.76 ± 0.09^a^	103.59 ± 1.81^a^	38.34 ± 0.03^a^
(°C)	38.31–38.5	89	4.56 ± 0.52^a^	15.41 ± 1.82^a^	33.77 ± 1.85^a^	14.81 ± 0.08^a^	102.24 ± 1.67^a^	38.37 ± 0.02^a^
	38.51–38.7	65	4.73 ± 0.61^a^	15.41 ± 2.07^a^	32.53 ± 1.87^a^	14.87 ± 0.10^a^	100.23 ± 1.96^a^	38.37 ± 0.03^a^
	>38.7	84	5.21 ± 0.55^a^	13.56 ± 1.88^a^	35.38 ± 1.79^a^	14.92 ± 0.09^a^	104.86 ± 1.78^a^	38.37 ± 0.03^a^
	*P*-value	-	0.7655	0.7911	0.7332	0.5426	0.325	0.7041

^1^DIM = days in milk; Cortisol_BS1 = cortisol concentration at blood sampling 1, Dopamine_BS1 = dopamine concentration at blood sampling 1, Serotonin_BS1 = serotonin at blood sampling 1, IgA_BS1 = immunoglobulin A at blood sampling 1, HR_BS1 = heart rate at blood sampling 1, RT_BS1 = rectal temperature at blood sampling 1

^2^logIgA = log-transformed immunoglobulin A, HR = heart rate at blood sampling 2 (beats/min), RT = rectal temperature at blood sampling 2 (°C)

^3^*P*-value = significance of fixed effects by F-statistics; Type III test of fixed effects

^a-c^significances of pairwise comparisons are indicated with different letters (*P* < 0.05)

**Table 5:** Least square means (LSM) with corresponding SE from model 4a for blood and physiological biomarkers and their association with milk yield class three days before blood sampling 2 (BS2).

Blood and physiological biomarkers^2^								
Fixed effect^1^	Effect class	N	Cortisol	Dopamine	Serotonin	logIgA	HR	RT
Herd	1	37	3.97 ± 0.74	16.88 ± 2.89^ab^	26.87 ± 2.70^c^	14.66 ± 0.11^b^	100.89 ± 2.44^bc^	38.35 ± 0.03
	2	75	5.39 ± 0.52	16.63 ± 1.84^ab^	34.69 ± 1.89^ab^	15.09 ± 0.08^a^	97.44 ± 1.70^c^	38.38 ± 0.02
	3	60	5.85 ± 0.59	17.80 ± 2.12^a^	29.74 ± 2.16^bc^	14.85 ± 0.09^b^	109.00 ± 1.95^a^	38.39 ± 0.03
	4	59	4.74 ± 0.58	11.36 ± 2.11^bc^	31.79 ± 2.13^bc^	14.92 ± 0.09^ab^	105.66 ± 1.92^ab^	38.39 ± 0.03
	5	85	4.32 ± 0.50	8.85 ± 1.78^c^	39.21 ± 1.83^a^	14.87 ± 0.08^b^	103.94 ± 1.22^b^	38.38 ± 0.02
	*P*-value^3^	-	0.1740	0.0034	0.0006	0.021	0.0002	0.8438
Parity	1	129	4.95 ± 0.43	14.90 ± 1.58	31.95 ± 1.58	14.84 ± 0.06	103.43 ± 1.43	38.40 ± 0.02
	2	103	5.16 ± 0.46	12.03 ± 1.64	30.61 ± 1.66	14.88 ± 0.07	101.85 ± 1.50	38.38 ± 0.02
	3	84	4.45 ± 0.52	15.98 ± 1.87	34.82 ± 1.88	14.91 ± 0.08	104.88 ± 1.70	38.36 ± 0.02
	*P*-value	-	0.5704	0.2099	0.2238	0.7576	0.3774	0.4803
DIM	70–120	69	4.28 ± 0.56	17.34 ± 2.10^a^	32.55 ± 2.05	15.06 ± 0.08	105.53 ±1.85	38.37 ± 0.26
	121–180	82	5.59 ± 0.51	12.72 ± 1.83^ab^	33.80 ± 1.84	14.85 ± 0.08	101.17 ± 1.66	38.37 ± 0.02
	181–239	73	4.99 ± 0.53	11.22 ± 1.89^b^	30.55 ± 1.93	14.82 ± 0.08	103.64 ± 1.75	38.40 ± 0.02
	≥240	92	4.55 ± 0.49	15.94 ± 1.73^ab^	32.94 ± 1.77	14.78 ± 0.07	103.20 ± 1.60	38.36 ± 0.02
	*P*-value	-	0.2920	0.0831	0.6567	0.0654	0.3524	0.6343
Milk Yield(kg)	Low ≤32.5	101	5.20 ± 0.50	13.81 ± 1.82	33.53 ± 1.82	14.95 ± 0.07	103.31 ± 1.64	38.39 ± 0.02
	Medium 32.5–40	114	4.79 ± 0.45	14.52 ± 1.61	33.52 ± 1.65	14.81 ± 0.07	103.82 ± 1.58	38.38 ± 0.02
	High >40	101	4.57 ± 0.48	14.59 ± 1.76	30.33 ± 1.76	14.87 ± 0.07	103.03 ± 1.58	38.36 ± 0.02
	*P*-value	-	0.6693	0.9392	0.3882	0.3076	0.9362	0.6743

^1^DIM = days in milk, Milk Yield = average of daily milk yield three days before blood sampling 2

^2^logIgA = log-transformed immunoglobulin A, HR = heart rate (beats/min), RT = rectal temperature (°C)

^3^*P*-value = significance of fixed effects by F-statistics; Type III test of fixed effects

^a-c^significances for pairwise comparisons are indicated with different letters; (*P* < 0.05)

**Fig. 4:**
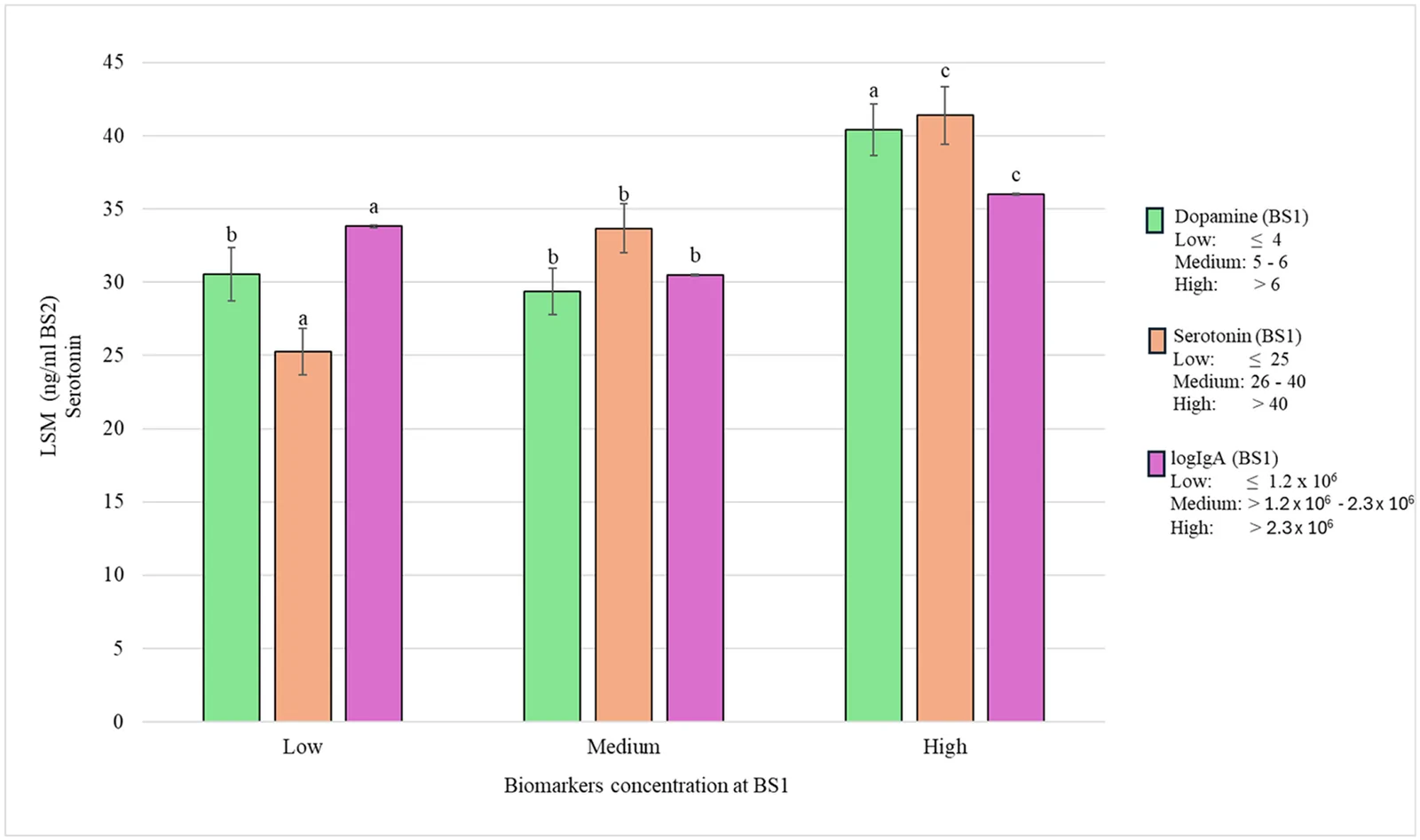
Least square means (LSM) for serotonin at blood sampling 2 (BS2; after behavior assessment) in dependency of dopamine, serotonin and logIgA concentrations at blood sampling1 (BS1; after calving). Different letters within classes of biomarkers indicate significant differences (*P* < 0.05).

Dopamine_BS1 (*P* < 0.001) and IgA_BS1 were significantly associated (*P* = 0.033) with the serotonin concentration at BS2. The LSM for the serotonin concentration (69.86 ± 5.90 ng/ml) at BS2 was significantly higher (*P* < 0.05) in cows with low cortisol concentrations at BS1 compared to cows with high cortisol concentrations at BS1 (12.39 ± 5.17 ng/ml) (Fig. 4). Very high IgA levels at BS1 were also associated with high LSM for serotonin concentrations at BS2 (LSM = 36.02 ± 1.59 ng/ml) (Fig. 4). Cows with low cortisol levels at BS1 showed high dopamine concentrations in BS2 (LSM = 25.72 ± 6.24 ng/ml). HR at BS1 had a significant (*P* < 0.001) influence on HR at BS2. Cows with low HR (≤ 88 bpm) at BS1 also had significantly lower LSM (*P* < 0.05) for HR at BS2 (94.98 ± 1.79 bpm). In analogy, high HR at BS1 (> 104 bpm) was associated with significantly higher LSM (*P* < 0.05) for HR at BS2 (109.96 ± 1.79 bpm).

Spearman correlation coefficients between blood and physiological biomarkers at BS1 and blood and physiological biomarkers at BS2 support the LSM results and are presented in the supplementary material S2, Table S2_3.

### Associations between milk production, milking behavior, and blood and physiological biomarkers at BS2

F-Statistics, LSM and significances of pairwise comparisons from model 4 are presented in Table 5 for MY, in Table 6 for KOD, in Table 7 for IMD and in Table 8 for NVD. Cortisol concentrations were higher in cows with a lower MY (LSM = 4.12 ± 0.50 ng/mL) than cows with a higher MY (LSM = 4.57 ± 0.48 ng/mL). Cows with lower milk production exhibited lower dopamine concentrations (LSM = 13.81 ± 1.82 ng/m) than high-yielding cows (LSM = 14.59 ± 1.76 ng/mL). Serotonin concentrations were higher in low-yielding (LSM = 33.53 ± 1.82 ng/mL) than in high-yielding cows (LSM = 30.33 ± 1.76 ng/mL). Similarly, RT was higher in low-yielding (LSM = 38.39 ± 0.02 ng/mL) than in high-yielding cows (LSM = 38.36 ± 0.02 ng/mL).

**Table 6:** Least square means (LSM) with corresponding SE from model 4b for blood and physiological biomarkers and their association with the average no. of kick-offs per day of the milking device in the automatic milking system three days before blood sampling 2 (BS2).

Blood and physiological biomarkers^2^								
Fixed effect^1^	Effect class	N	Cortisol	Dopamine	Serotonin	logIgA	HR	RT
Farm	1	36	3.79 ± 0.86	10.10 ± 4.43	23.61 ± 2.51^a^	14.63 ± 0.16^a^	96.43 ± 3.71	38.38 ± 0.04
	4	56	4.48 ± 0.65	6.25 ± 3.10	30.67 ± 1.87^b^	15.00 ± 0.12^b^	101.27 ± 2.77	38.40 ± 0.03
	*P*-value^3^	-	0.3802	0.3390	0.0026	0.0136	0.1543	0.5824
Pairty	1	129	4.10 ± 0.71	11.32 ± 3.46	29.79 ± 2.05	14.84 ± 0.13	98.14 ± 3.03	38.40 ± 0.03
	2	103	4.53 ± 0.97	3.27 ± 4.78	24.84 ± 2.82	14.89 ± 0.18	97.03 ± 4.18	38.42 ± 0.05
	3	84	3.77 ± 0.88	9.93 ± 4.44	26.80 ± 2.55	14.71 ± 0.16	101.37 ± 3.77	38.34 ± 0.04
	*P*-value	-	0.7617	0.2105	0.1884	0.5953	0.5806	0.2429
DIM	70–120	69	3.29 ± 0.90	15.16 ± 4.90^a^	27.35 ± 2.62	14.54 ± 0.17^b^	98.25 ± 3.88	38.34 ± 0.04
	121–180	82	4.22 ± 1.04	0.94 ± 5.10^b^	27.19 ± 3.00	14.94 ± 0.19^ab^	96.63 ± 4.44	38.42 ± 0.05
	181–239	73	4.59 ± 0.91	6.91 ± 4.42^ab^	26.39 ± 2.65	14.98 ± 0.17^a^	102.29 ± 3.92	38.39 ± 0.04
	≥240	92	4.45 ± 0.85	9.69 ± 4.08^ab^	27.64 ± 2.47	14.79 ± 0.16^ab^	98.21 ± 3.65	38.40 ± 0.04
	*P*-value	-	0.5996	0.1184	0.9769	0.1153	0.6412	0.4060
KOD	Low 0	75	4.33 ± 0.41	15.48 ± 2.08	29.40 ± 1.20	14.98 ± 0.07	104.07 ± 1.77	38.36 ± 0.02
	Medium 0–0.5	9	4.56 ± 1.26	5.71 ± 6.47	29.26 ± 3.66	14.85 ± 0.06	96.50 ± 5.42	38.36 ± 0.06
	High >0.5	8	3.52 ± 1.35	3.33 ± 6.43	22.77 ± 3.92	14.80 ± 0.07	95.97 ± 5.80	38.45 ± 0.06
	*P*-value	-	0.8216	0.0890	0.2619	0.6214	0.1974	0.3874

^1^DIM = days in milk, KOD = average number of kick-offs per day against the milking device during milking within three days before blood sampling 2

^2^logIgA = log-transformed immunoglobulin A, HR = heart rate (beats/min), RT = rectal temperature (°C)

^3^*P*-value = significance of fixed effects by F-statistics; Type III test of fixed effects

^a-c^significances for pairwise comparisons are indicated with different letters; (*P* < 0.05)

**Table 7:** Least square means (LSM) with corresponding SE from model 4c for blood and physiological biomarkers and their association with the average number of incomplete milkings per day in the automatic milking system three days before blood sampling 2 (BS2).

Blood and physiological biomarkers^2^								
Fixed effect^1^	Effect class	N	Cortisol	Dopamine	Serotonin	logIgA	HR	RT
Farm	1	36	3.42 ± 0.88	10.80 ± 4.34	24.80 ± 2.60^a^	14.56 ± 0.17^a^	97.90 ± 3.83	38.43 ± 0.04
	4	56	4.27 ± 0.69	5.91 ± 3.37	31.37 ± 2.03^b^	14.91 ± 0.13^b^	102.51 ± 2.99	38.45 ± 0.03
	*P*-value^3^	-	0.2845	0.2219	0.0057	0.0171	0.1795	0.6649
Parity	1	129	4.06 ± 0.76	11.48 ± 3.73	30.93 ± 2.25	14.76 ± 0.14	99.62 ± 3.31	38.44 ± 0.04
	2	103	4.15 ± 1.03	2.93 ± 5.04	26.02 ± 3.04	14.81 ± 0.19	98.25 ± 4.47	38.48 ± 0.05
	3	84	3.34 ± 0.86	10.65 ± 4.24	27.29 ± 2.54	14.63 ± 0.16	102.74 ± 3.74	38.39 ± 0.04
	*P*-value	-	0.6697	0.1750	0.2018	0.5850	0.5793	0.1596
DIM	70–120	69	3.02 ± 0.87	17.00 ± 4.51^a^	28.00 ± 2.57	14.46 ± 0.16^b^	99.86 ± 3.79	38.37 ± 0.04
	121–180	82	3.95 ± 1.08	0.21 ± 5.48^b^	28.36 ± 3.20	14.84 ± 0.20^ab^	98.18 ± 4.72	38.48 ± 0.05
	181–239	73	4.25 ± 0.89	7.66 ± 4.28^ab^	27.25 ± 2.62	14.90 ± 0.17^a^	103.97 ± 3.86	38.44 ± 0.04
	≥240	92	4.17 ± 0.95	8.54 ± 4.57^ab^	28.72 ± 2.79	14.73 ± 0.18^ab^	98.82 ± 4.11	38.46 ± 0.04
	*P*-value	-	0.6339	0.0655	0.9663	0.1300	0.5790	0.1995
IMD	Low 0	71	4.48 ± 0.42	15.80 ± 2.18	29.30 ± 1.24	14.80 ± 0.08	103.95 ± 1.83	38.35 ± 0.02
	Medium 0–1	16	3.25 ± 1.01	8.18 ± 4.97	27.34 ± 2.97	14.86 ± 0.19	98.24 ± 4.38	38.40 ± 0.05
	High >1	5	3.81 ± 1.66	1.07 ± 7.98	27.61 ± 4.91	14.54 ± 0.31	98.43 ± 7.23	38.56 ± 0.08
	*P*-value	-	0.5076	0.1187	0.8036	0.6633	0.4114	0.0250

^1^DIM = days in milk, ^3^IMD = average number of incomplete milkings per day within three days before blood sampling 2

^2^logIgA = log-transformed immunoglobulin A, HR = heart rate (beats/min), RT = rectal temperature (°C)

^3^*P*-value = significance of fixed effects by F-statistics; Type III test of fixed effects

^a-c^significances for pairwise comparisons are indicated with different letters; (*P* < 0.05)

**Table 8:** Least square means (LSM) with corresponding SE from model 4d for blood and physiological biomarkers and their association with the number of milking visits per day within three days before blood sampling 2 (BS2).

Blood and physiological biomarkers^2^								
Fixed effect^1^	Effect class	N	Cortisol	Dopamine	Serotonin	logIgA	HR	RT
Farm	1	37	3.73 ± 0.74^b^	16.93 ± 2.82^a^	26.23 ± 2.71^c^	14.62 ± 0.11^c^	100.78 ± 2.44^bc^	38.34 ± 0.03
	2	75	5.37 ± 0.52^ab^	16.30 ± 1.84^a^	34.31 ± 1.90^b^	15.11 ± 0.08^a^	97.61 ± 1.71^c^	38.38 ± 0.02
	3	60	5.76 ± 5.76^a^	17.56 ± 2.12^a^	29.25 ± 2.15^bc^	14.84 ± 0.09^bc^	109.32 ± 1.94^a^	38.40 ± 0.03
	4	59	4.67 ± 4.67^ab^	11.59 ± 2.11^ab^	31.90 ± 2.16^bc^	14.90 ± 0.09^ab^	105.22 ± 1.94^ab^	38.38 ± 0.03
	5	85	4.54 ± 4.54^ab^	8.99 ± 1.85^b^	39.56 ± 1.90^bc^	14.90 ± 0.08^ab^	104.35 ± 1.71^ab^	38.39 ± 0.02
	*P*-value^3^	-	0.2006	0.0073	0.0003	0.0073	0.0001	0.7813
Parity	1	129	5.03 ± 0.41	14.69 ± 1.48	32.58 ± 1.49	14.84 ± 0.06	103.75 ± 1.35	38.41 ± 0.02
	2	103	5.09 ± 0.45	12.11 ± 1.61	30.27 ± 1.64	14.88 ± 0.07	101.85 ± 1.48	38.38 ± 0.02
	3	84	4.33 ± 0.49	16.02 ± 1.79	33.91 ± 1.80	14.91 ± 0.07	104.76 ± 1.62	38.35 ± 0.02
	*P*-value	-	0.4443	0.2294	0.2985	0.7319	0.3824	0.1630
DIM	70–120	69	4.29 ± 0.56	17.36 ± 2.07^a^	31.88 ± 2.04	15.08 ± 0.08^a^	105.95 ± 1.83	38.37 ± 0.03
	121–180	82	5.47 ± 0.50	12.56 ± 1.83^ab^	33.20 ± 1.84	14.85 ± 0.08^b^	101.13 ± 1.66	38.37 ± 0.02
	181–239	73	5.01 ± 0.53	11.29 ± 1.87^b^	30.75 ± 1.92	14.81 ± 0.08^b^	103.77 ± 1.73	38.40 ± 0.02
	≥240	92	4.50 ± 0.48	15.88 ± 1.72^ab^	33.17 ± 1.77	14.76 ± 0.07^b^	102.97 ± 1.59	38.36 ± 0.02
	*P*-value	-	0.3618	0.0807	0.7583	0.0271	0.2689	0.2366
NVD	Low ≤2.5	90	5.18 ± 0.50	13.30 ± 1.78	32.14 ± 1.82	14.98 ± 0.07	104.92 ± 1.64	38.41 ± 0.02
	Medium 2.5–3.5	118	5.08 ± 0.42	16.07 ± 1.56	33.78 ± 1.54	14.85 ± 0.06	102.81 ± 1.39	38.36 ± 0.02
	High >3.5	108	4.18 ± 0.47	13.44 ± 1.67	30.83 ± 1.70	14.80 ± 0.07	102.64 ± 1.53	38.37 ± 0.02
	*P*-value	-	0.2439	0.3742	0.4176	0.207	0.5454	0.2366

^1^DIM = days in milk, NVD = the average number of milking visits per day within three days before blood sampling 2

^2^logIgA = log-transformed immunoglobulin A, HR = heart rate (beats/min), RT = rectal temperature (°C)

^3^*P*-value = significance of fixed effects by F-statistics; Type III test of fixed effects

^a-c^significances of pairwise comparisons are indicated with different letters; (*P* < 0.05)

Higher cortisol concentrations were observed in cows with a low frequency for KOD (LSM = 4.33 ± 0.41 ng/mL) compared to cows with a high frequency for KOD (LSM = 3.52 ± 1.35 ng/mL). Dopamine concentrations were higher in cows with fewer KOD (LSM = 15.48 ± 2.08 ng/mL) than in cows with more frequent KOD (LSM = 3.33 ± 6.43 ng/mL). Serotonin levels were also higher in cows with fewer KOD (LSM = 29.40 ± 1.20 ng/mL) than in cows with frequent KOD (LSM = 22.77 ± 3.92 ng/mL). LogIgA was also higher in cows with fewer KOD (LSM = 14.98 ± 0.08 mg/mL) than in cows with frequent KOD (LSM = 14.80 ± 0.07 mg/mL). Similarly, HR was higher in cows with fewer KOD (LSM = 104.07 ± 1.78 bpm) than in cows with frequent KOD (LSM = 95.97 ± 2 bpm). However, cows with frequent KOD tended to have a higher RT (LSM = 38.36 ± 0.02 °C vs. 38.45 ± 0.07 °C).

Cortisol levels tended to be higher in cows with fewer IMD (LSM = 4.49 ± 0.42 ng/mL) than in those with more frequent IMD (LSM = 3.81 ± 1.66 ng/mL). There was also a tendency for higher dopamine concentrations in cows with fewer IMD (LSM = 15.80 ± 2.18 ng/mL) compared with cows with more IMD (LSM = 1.07 ± 7.98 ng/mL). Serotonin levels were higher in cows with fewer IMD (LSM = 29.30 ± 1.24 ng/mL) than in those with more (LSM = 27.61 ± 4.91 ng/mL). LogIgA concentrations were higher in cows with fewer IMD (LSM = 14.80 ± 0.08 mg/mL) than in cows with more IMD (LSM = 14.54 ± 0.31 mg/mL). HR tended to be higher in cows with fewer IMD (LSM = 103.95 ± 1.83 bpm) than in cows with more IMD (LSM = 98.43 ± 7.23 bpm). RT was significantly affected by the frequency of IMD (*P* = 0.0250): cows experiencing more frequent IMD showed higher RT (LSM = 38.56 ± 0.08 °C), compared with cows experiencing fewer IMD (LSM = 38.35 ± 0.02 °C).

Cortisol concentrations were higher in cows with NVD ≤ 2.5 (LSM = 5.19 ± 0.50 ng/mL) compared to cows with NVD > 3.5 (LSM = 4.18 ± 0.45 ng/mL). HR was higher in cows with lower visit frequency (104.92 ± 1.64 vs. 102.64 ± 1.54 bpm). RT also tended to be higher in cows with lower visit frequency (38.41 ± 0.02 vs. 38.37 ± 0.02 °C). All the above-mentioned LSM differences were non-significant (*P* > 0.05), and in consequence, should be interpreted with caution.

## Discussion

### Biomarkers in response to various stress conditions

In the ongoing discussions, we differentiated between acute and chronic stress in dairy cows.

Acute stress is generally described as a short-term physiological response to challenges, involving activation of the hypothalamic–pituitary–adrenal (HPA) axis and the sympathetic nervous system, and is associated with transient increases in heart rate and glucocorticoid (e.g., cortisol) secretion (Weber et al., 2022). Acute stress is a short-term reaction (lasting from a few seconds to a few minutes) triggered by specific events, accompanied by typical physiological changes. Consequently, in the present study, we considered stressful calving and human behavior tests as acute stressors. In contrast, chronic stress implies prolonged or repeated exposure to stressors, which may result in dysregulation of HPA axis activity, altered stress reactivity, and sustained inflammatory processes (Rohleder et al., 2019). In such context, and according to Behren et al. (2023), we related chronic stress to milk production and the behavioral responses in the technical AMS environment.

Regarding stress definitions, observational studies have inherent limitations in establishing causality (MacLeod et al., 2002). Furthermore, the concept of “stress” is multifactorial and context-dependent (Allen et al., 2014). Nevertheless, the conceptual distinction between acute and chronic stress is widely used in stress physiology. This framework enables a structured interpretation of physiological responses in terms of short-term adaptation versus potential longer-term dysregulation (Trevisi et al., 2009).

### Biomarker responses in relation to calving associated stress conditions

Cortisol concentrations at BS1 (4.74 ng/mL) were low, indicating a mild stress response due to calving. It should be noted that the physiological response measured at BS1 (0–14 days postpartum) likely reflects the combined effects of the calving event together with concurrent management and environmental changes occurring during this period, including regrouping, altered social interactions, dietary transition and the onset of the milking routine, rather than the calving event in isolation. For brevity, this combined response is hereafter referred to as “calving stress”. The pilot study (see supplementary material S1) involved sampling cows after routine claw trimming. Claw trimming is a pronounced acute stressor for cows (Koenneker et al., 2023), implying substantially higher cortisol levels (28.31 ng/mL) than observed after calving in the present study. Elevated cortisol due to the physiological demands of calving were identified in previous studies, justifying the classification of calving as an acute stressor in HF cattle (Jacob et al., 2001; Sano et al., 2023). In the present study, the interval between calving and blood sampling had a significant effect on cortisol levels: cows sampled at the calving date showed higher concentrations than cows sampled 14 days postpartum (*P* < 0.001). Sampling occurred in the early afternoon due to logistical constraints rather than during the generally early-morning cortisol peak. However, acute stress responses were still clearly detectable. Consistently, Albanat et al. (2013) reported cortisol peaks at birth (6.5 ng/mL), but returns to baseline (2 ng/mL) within 12 hours postpartum.

Dopamine concentrations at BS1 (6.67 ng/mL) after calving were substantially lower than the concentrations (12.07 ng/mL) in the pilot claw trimming study (see supplementary material S1). Results suggest that calving did not trigger a significant acute stress response, i.e. increased dopamine release, as indicated by Snider et al. (1983) and Caceres et al. (2023). Serotonin concentrations at BS1 (43.65 ng/mL) were higher than those in the pilot claw trimming study (20.51 ng/ml), supporting the interpretation that the cows were not exposed to excessive stress after calving. Serotonin is generally considered as a marker for well-being, with reduced levels typically associated with stress (Caceres et al., 2023). Nevertheless, serotonin responses due to stressors are reported inconsistently in the literature. Depending on the genetic background and the characteristics of the stressor, blood and platelet serotonin levels may increase or decrease. Both elevated and reduced levels have been proposed as indicators of stress (Ghosh and Guha, 2023).

Physiological markers aligned with the cortisol patterns, displaying similar responses for HR and RT. Accordingly, close correlations between HR and RT were reported under heat stress conditions (Gaughan et al., 2012; Rejeb et al., 2016). RT adjustments were mostly observed after prolonged stress, while HR reacted more rapidly to stressors (Brown-Brandl et al., 2005). Overall, both HR and RT showed only minimal and transient alterations, indicating limited physiological impact due to calving.

### Biomarker responses in relation to human handling stress conditions

Cortisol concentrations at BS2 (4.94 ng/mL) were lower than those in the pilot claw trimming study (28.31 ng/mL), mirroring the patterns observed at BS1 (see supplementary material S1). Such finding indicates that the cows were not excessively stressed after the behavioral assessments. In agreement, routine human contact, such as veterinary examinations or artificial insemination, were not significantly associated with blood cortisol concentrations in dairy cattle (Rushen et al., 2001; Koenneker et al., 2023; Ferreira et al., 2025). In contrast, short-term environmental or logistical stressors such as transport can transiently elevate cortisol concentrations ranging from 18.1 to 43.5 ng/mL, which typically return to the baseline within a few hours (Gebresenbet et al., 2012; Lay et al., 1992). Serotonin concentrations at BS2 (33.44 ng/mL) were higher than in the pilot claw trimming study (20.51ng/mL), supporting the fact that cows were not exposed to excessive stress due to human handling.

Heart rates significantly increased with increasing TTI1 at BS2. Afterwards, on the same day, the effect of TTI2 on HR was non-significant. Such findings, i.e., response in a more relaxed manner, might be explained with a memory effect. In our study, cows displaying a lower HR had significantly higher RT at BS2. This was surprising, but LSM differences for RT comprised only 0.08 °C. Furthermore, HR and RT reflect different physiological regulatory systems and may not necessarily change in parallel under field conditions (Becker et al., 2020). Fieguth (2014) reported increased HR following human contact, for example during claw care, whereas repeated gentle handling induced adaptation and reduced HR (Waiblinger et al., 2003). We also expected a significant effect of AD on the biomarker responses, but AD had no significant influence on any biomarker in this study. This finding suggests that cows were accustomed to human contact. Similarly, Waiblinger et al. (2003) emphasized only weak relationships between AD and short-term physiological stress responses. Sharma et al. (2019) described AD as a behavioral indicator of welfare and showed associations with dirtiness and health disorders.

### Biomarkers in response to different stressors: simultaneous assessment of calving stress and human handling

In this study, we evaluated whether selected biomarkers respond differently to calving stress and behavior tests. For all interpretations, the long interval between the consecutive measurement dates (average of 1818 days) should be considered. Dopamine and serotonin levels showed similar intra-individual pattern across both sampling points, indicating limited fluctuation over time. We found a significant favorable association between both neurotransmitters at each sampling point and overtime, generally indicating that cows with lower dopamine concentrations also had lower serotonin levels, whereas higher dopamine coincided with higher serotonin. Such patterns reflect the close functional coupling of the two neurotransmitters, as previously reported in rats (Kang et al., 2023). Similarly, Fischer et al. (2017) emphasized the synergistic effect of dopamine and serotonin in modulating reward and learning. Furthermore, genetic associations have been described between dopamine (*DRD2/DRD3*) and serotonin receptor (*HTR2A*) genes, and with behavior and temperament in beef cattle (Garza-Brenner et al., 2017). However, relationships between both biomarkers might differ, depending on the environmental conditions. For example, Holstein cows kept in comfort housing conditions exhibited increased serotonin but decreased dopamine levels (Cáceres et al., 2023).

In line with neurotransmitter associations, IgA at BS1 was significantly associated with the serotonin concentration at BS2 in the present study. This is a first hint for similar responses of same cows to different stressors, but it is imperative to verify such findings in recording schemes with closer sampling intervals. The observed association between IgA and serotonin concentrations indicates the synergies of dopaminergic and serotonergic systems in mammals and the role of IgA as a stress-sensitive immune marker.

Regarding IgA dynamics, concentrations at BS1 and BS2 did not differ significantly, indicating that cows with higher IgA at BS1 also tended to have higher IgA at BS2, and vice versa. These patterns reflect the stability of IgA levels over the two sampling time points. In contrast, IgA concentrations in the pilot claw trimming study (see supplementary material S1) were markedly lower. Lower values might reflect acute stress responses, potentially mediated by the activation of the hypothalamic–pituitary–adrenal (HPA) axis and subsequent changes in cortisol levels. Although stress-induced cortisol increases are often associated with decreases in salivary IgA, these responses varied depending on the type of sample, the tissue and the body site (Lara-Padilla et al., 2015; Kurimoto et al., 2016; Lasisi et al., 2017). Consistently, cows with lower cortisol at BS1 also displayed lower IgA levels, even though IgA levels are generally expected to decline when cortisol levels rise (Kugler et al., 1991). Results highlight the context-dependent relationship between neuroendocrine activity and IgA, indicating that IgA may be a sensitive indicator of stress in certain situations. IgA concentrations also vary physiologically around calving, independent of stress exposure. Guidry et al. (1980) reported a peak for serum IgA levels before calving, a decline postpartum and fluctuations throughout lactation.

For HR, the results of our study showed a significant relationship between HR at BS1 and HR at BS2, indicating that cows with higher HR due to calving stress at BS1 show higher HR due to human handling tests at BS2. Such association pattern reflects a consistent physiological response due to both stressors, and is consistent with the stability observed for most biomarkers. The mean HR at BS1 (101.1 ± 15.21 bpm) and BS2 (103.15 ± 15.03 bpm) were both above the 60–90 bpm reference range reported by Janzekovic et al. (2010). Such HR elevation may be due to mild stress caused by blood sampling and the presence of an unfamiliar person.

### Biomarker responses in relation to production stress

Dairy cows typically experience the strongest physiological load in the early lactation period, followed by a subsequent decline with progressing days in milk. The early lactation period is characterized by elevated inflammatory markers, higher cortisol levels and a negative energy balance, but Yehia et al. (2021) indicated adaptive mechanisms with progressing time. In such context, Davis et al. (2023) found higher stress levels in first parity than multiparous cows, supporting the results from the present study.

In the present study, MY showed no significant effect on cortisol concentrations. Interestingly, cows with lower MY displayed higher cortisol levels than the high-yielding cow group, indicating that high milk production per se does not trigger physiological stress. Instead, cows with inadequate physiological adaptation may express lower MY and higher cortisol. This aligns with the findings of Marçal-Pedroza et al. (2023), i.e., elevated cortisol levels accompanied with impaired productivity in temperamental and reactive animals. On the other hand, elevated cortisol can reduce blood flow to the mammary gland, limiting glucose availability and inhibiting oxytocin release and action, ultimately impairing milk production (Bruckmaier and Blum, 1998; Wellnitz et al., 2001; Bruckmaier, 2005; Hall et al., 2020). Similarly, cows with higher milk production exhibited lower HR, likely reflecting their increased energy demands (Janzekovic et al., 2010).

### Biomarker responses in relation to behavior in a technical environment

Behavior during milking was assessed by analyzing the frequency of kicks against the milking equipment. Cows with fewer KOD exhibited higher concentrations of cortisol, dopamine, serotonin and IgA, as well as higher HR. However, by trend, increased KOD implied slightly elevated RT. Marçal-Pedroza et al. (2023) suggested an increased KOD frequency as a suitable stress indicator for milking behavior in a technical environment, because frequently kicking cows displayed 28.4 % higher milk cortisol levels than non-kicking cows. The present study revealed an opposite trend: calmer cows with fewer KOD showed stronger physiological stress responses. As a possible explanation, we can only speculate that highly anxious or chronically stressed cows remain immobile in the milking robot and exhibit minimal kicking behavior. Hence, KOD frequency may not fully capture a cows’ stress status, highlighting the importance of integrating several behavioral and physiological indicators when assessing welfare in automated milking systems (Yin et al., 2019).

Behavioral responses during milking were also evaluated based on IMD. Like the pattern observed for KOD, IMD had no significant effect on physiological or blood biomarkers. However, cows with fewer IMD exhibited higher concentrations of cortisol, dopamine, serotonin and IgA, as well as elevated heart rates. In contrast, cows with more IMD showed slightly higher RT, confirming the complexity and varying responses of different biomarkers in a technical environment as outlined by Schwanke et al. (2022).

In the present study, cows entering the AMS less frequently displayed higher cortisol and IgA concentrations, as well as elevated HR and RT. Helmreich et al. (2016) reported no significant effects of milking frequency on cortisol or IgA, while Schwanke et al. (2022) found that more fearful cows consumed less concentrate from the AMS due to fewer visits.

Cows entering the AMS less frequently displayed higher cortisol and IgA concentrations, as well as elevated HR and RT.

### Study limitations and respective concerns

For cortisol, dopamine, serotonin and IgA, no universally accepted biological or clinical cut-off values have been established for dairy cows. Reported concentrations varied considerably among studies due to differences in biological matrices, analytical methods, sampling time, stage of lactation, management conditions and population characteristics, and biologically meaningful thresholds for these biomarkers do not exist (e.g., Caceres et al., 2023; Ramalan et al., 2024). Consequently, as a basis for interpretations of biomarker responses to the possible stressors (calving, human handling, milk production) in the present study population, we created a pronounced acute stress situation via claw trimming. However, the sample size for the cows considered in this pilot study was small, and respective results are only of preliminary nature.

In such context, reliable biological thresholds when defining classes of explanatory biomarker variables in mixed model analyses, are missing. We created biomarker effect classes considering equal sample sizes, following quantile approaches in genetic association studies (e.g., Weigel, 2017). From a statistical perspective, there are severe concerns when categorizing continuous explanatory variables in association analyses: information loss because all variables from the same class are treated equally, loss of power due to the reduction of overall variance, and two individuals with almost same response traits might be allocated to different classes. Furthermore, quantile-based categorization of continuous biomarkers might reduce statistical power and obfuscate dose-response relationships.

Advantages for classifications are practical association illustrations in case of non-linear relationships between response variables and effects, and ongoing applications in animal breeding. Selection response calculations depend on phenotypic differences between created sub-groups of the population, determining selection intensity. Consequently, for ongoing assessments in a breeding context, LSM for biomarkers in distinct categories are helpful to evaluate the potential of genetic selection.

In the association analyses, we fitted multiple related models to infer the numerous effects on explanatory variables on dependent traits. However, such modelling approach may inflate the overall Type I error, and residual inflation of false-positive findings across the entire analytical framework cannot be completely ruled out.

A last concern is related to large interval for consecutive measurements of the same cow (average: 181 days). Especially in the context of cow behavior responses, a dense longitudinal data structure (ideally daily) is needed to infer reliable covariance components among traits, and for proper corrections of disturbing environmental effects. Such ideal research design was recently emphasized by Behren et al. (2026) for behavior trait responses, but on-farm research might be hampered due to practical constraints and implies tremendous logistic efforts.

## Conclusion

This study investigated physiological, neuroendocrine and behavioral responses of dairy cows to various stressors, including calving (reflecting the combined effects of the calving event and concurrent postpartum management changes), human interactions and the technical milking robot environment. Cortisol concentrations indicated only moderate acute stress responses after calving and human handling. Comparisons with a more intensive stressor (claw trimming) suggest that routine management procedures (i.e., calving, human handling) induce limited physiological disruption. Dopamine and serotonin exhibited consistent intra- and inter-individual patterns, suggesting a functional coupling between these both neurotransmitters. IgA concentrations demonstrated stability across sampling points, supporting temporal stability of this immune-related biomarker under field conditions. Behavioral indicators from the milking robot system indicated complex associations with physiological measures. Lower frequencies of kicking behavior were related with higher concentrations of stress-related biomarkers, suggesting that behavioral responses alone may not reliably reflect physiological stress states in AMS. Milk production was not directly associated with elevated stress responses, indicating that high milk yield is not necessarily linked to physiological strain under the present farming conditions.

## Ethics declaration

This study was conducted in accordance with the following guidelines for animal welfare and/or reporting: German Animal Welfare Act. This study was approved by the State government of North Rhine-Westphalia, Germany. (Approval No. 81-02.04.40. 2023.VG082).

## CRediT authorship contribution statement

**Laura Breitmeier:** Data curation, Formal analysis, Investigation, Methodology, Visualization, Writing – original draft. **Kerstin Brügemann:** Formal analysis, Data curation. **Gerhard Schuler:** Methodology. **Larissa Behren:** Data curation. **Julia Stuhlträger:** Writing – review & editing. **Frank Rosner:** Data curation. **Hermann Swalve:** Writing – review & editing, Validation, Conceptualization. **Sven König:** Writing – review & editing, Validation, Resources, Funding acquisition, Conceptualization. **Katharina May:** Project administration, Conceptualization.

## Declaration of competing interest

We declare that we have no conflicts of interest.

## Data Availability

The data is available from the corresponding author upon reasonable request.

## Appendix Supplementary materials

Image, application 1

Image, application 2
