# Supplementary material for: Inferring associations among behavior, hormonal, neurotransmitter and IgA responses of dairy cows housed in automatic milking systems
Source: Vet Anim Sci. 2026 Sep 5;34:100857. doi: 10.1016/j.vas.2026.100857 (PMC13573191; doi:10.1016/j.vas.2026.100857)
Supplement: Supplementary file 1 [file mmc1.docx]

**Supplementary material S1**

Pilot study: Comparisons of blood and physiological biomarkers after calving, after behavior assessment and under extreme stress conditions

**Preface**
Ten cows from one farm were randomly selected to represent a specific stress group to measure concentrations of cortisol, dopamine, serotonin and IgA in a particularly stressful situation, i.e., during fixation in a claw care crush after claw trimming. The data of the stress group served as a reference for biomarkers under extreme stressful conditions, because of the lack of established physiological reference ranges for stress biomarkers in dairy cows.

**Statistical analysis**
Differences for the same traits between BS1, BS2 and the stress reference group were tested via Wilcoxon rank-sum test for all traits using SAS OnDemand for Academics (SAS Institute Inc., Cary, NC, USA). All comparisons were performed individually in separate pairwise analyses, i.e., BS1 vs. BS2, BS1 vs. stress group, BS2 vs. stress group.

**Results**

Descriptive statistics for blood and physiological biomarkers at BS1, BS2 and at claw trimming crush (= reference stress group) are presented in the following Table A. In Table A, also results of the Wilcoxon rank-sum test for blood and physiological biomarkers at BS1, BS2 and at the claw-trimming crush are presented.

**Table A.** Descriptive statistics for biomarkers of cows at blood sampling 1 (BS1), blood sampling 2 (BS2) and for the stress group

| Biomarkers^1^ | BS1 | | BS2 | | Stress group | |
| --- | --- | --- | --- | --- | --- | --- |
|  | Median | (Min-Max) | Median | (Min-Max) | Median | (Min-Max) |
| Cortisol (ng/ml) | 3.29 | (0.30-28.76) | 3.59 | (0.30-29.03) | 36.75 | (6.93-40.00) |
| Dopamine (ng/ml) | 5.04 | (0.89-112.98) | 5.16 | (0.19-112.10) | 8.09 | (2.01-18.98) |
| Serotonin (ng/ml) | 30.76 | (3.21-665.50) | 31.16 | (2.68-183.45) | 12.28 | (9.89-79.25) |
| IgA (ng/ml) | 1.73x10^6^ | (1.4x10^5^-1.12x10^8^) | 2.7x10^6^ | (4.12x10^5^-1.39x10^7^) | 1.345x10^6^ | (9.27x10^5^-1.67x10^6^) |

^1^IgA = immunoglobulin A

The median for the cortisol concentration in the reference stress group was 36.75 ng/mL, which was significantly higher than the concentration at BS1 (median: 3.29 ng/mL) (P < 0.001) and BS2 (median: 3.59 4ng/mL) (P < 0.001). The difference in cortisol concentrations between BS1 and BS2 was not significant (P = 0.236). Dopamine concentrations for the median significantly (P < 0.001) differed between BS1 (median: 5.04: ng/mL) and BS2 (median: 5.16 ng/mL) when compared to the stress group (median: 8.09 ng/ml). No significant difference (P = 0.571) between BS1 and BS2 was found for the serotonin concentrations of 30.76 ng/mL and 31.16 ng/mL for the medians, respectively. Differences were significant (P < 0.001) between the stress group with a median concentration of 12.28 ng/mL and BS1, and between the stress group and BS2. The IgA concentrations differed significantly (P < 0.001) between BS1 (median: 1.73x106 ng/mL) and BS2 (median: 2.70 x106 ng/ml), and between BS2 and the stress group (median: 1.34x106). No significant difference for IgA concentrations was found between BS1 and the stress group.

**Remarks**

The stress group consisted of 10 cows and was exposed to an extremely stressful situation in a claw-care crush following claw trimming. These cows exhibited moderately elevated cortisol levels and decreased serotonin concentrations, indicative for standard acute stress responses (Koenneker et al. 2023; Caceres et al. 2023). This group was established to generate a reference for biomarker values, given the lack of established physiological reference ranges for cortisol, dopamine, serotonin and IgA in dairy cows. For animal welfare reasons, blood samples could only be taken from 10 animals in an extremely stressful situation. Thus, the stress group was only used in the Wilcoxon rank-sum test to enable a rough comparison of biomarker levels under various stress conditions, but was not considered in generalized linear mixed model analyses.

**References**

Caceres, S., J. Moreno, B. Crespo, G. Silvan, and J. C. Illera. 2023. Physiological stress responses in cattle used in the Spanish rodeo. Animals 13: 2654. https://doi.org/10.3390/ani13162654.

Koenneker, K., M. Schulze, L. Pieper, M. Jung, M. Schmicke, and F. Beyer. 2023. Comparative assessment of the stress response of cattle to common dairy management practices. Animals 13: 2115. https://doi.org/10.3390/ani13132115.
