# Supplementary material for: Inferring associations among behavior, hormonal, neurotransmitter and IgA responses of dairy cows housed in automatic milking systems
Source: Vet Anim Sci. 2026 Sep 5;34:100857. doi: 10.1016/j.vas.2026.100857 (PMC13573191; doi:10.1016/j.vas.2026.100857)
Supplement: Supplementary file 2 [file mmc2.docx]

**Supplementary material S2**

**Table S2_1: Correlations between blood and physiological biomarkers after calving at BS1** (additional results from the chapter “Associations between blood and physiological biomarkers after calving at BS1”)

| **BS1** | | | | | | | |
| --- | --- | --- | --- | --- | --- | --- | --- |
| **BS1** |  | Cortisol | Dopamin | Serotonin | logIgA | HR | RT |
|  | Cortisol | **1.00** |  |  |  |  |  |
|  | Dopamin | 0.08 | **1.00** |  |  |  |  |
|  | Serotonin | 0.02 | *** 0.37 | **1.00** |  |  |  |
|  | logIgA | 0.06 | -0.07 | 0.09 | **1.00** |  |  |
|  | HR | * 0.12 | 0.05 | 0.05 | 0.05 | **1.00** |  |
|  | RT | 0.08 | 0.01 | -0.01 | -0.05 | ** 0.14 | **1.00** |

**BS1** = blood sampling after calving; **logIgA** = log-transformed immunoglobulin A; **RT** = rectal temperature; **HR** = heart rate

*Significance of Spearman correlation coefficients: *P ≤ 0.05 (highlighted in blue); **P ≤ 0.01 (highlighted in green); ****P ≤ 0.001* (highlighted in violet)

**Table S2_2: Correlations between blood and physiological biomarkers after behavior assessment at BS2** (additional results from the chapter “Associations among blood biomarkers, physiological biomarkers and behavior traits after behavior assessments at BS2”)

| **BS2** | | | | | | | | | | | |
| --- | --- | --- | --- | --- | --- | --- | --- | --- | --- | --- | --- |
|  |  | Cortisol | Dopamin | Serotonin | logIgA | HR | RT | TTI 1 | TTI 2 | AD 1 | AD 2 |
| **BS2** | Cortisol | **1.00** |  |  |  |  |  |  |  |  |  |
|  | Dopamin | * 0.14 | **1.00** |  |  |  |  |  |  |  |  |
|  | Serotonin | 0.04 | *** 0.32 | **1.00** |  |  |  |  |  |  |  |
|  | logIgA | -0.01 | -0.10 | 0.07 | **1.00** |  |  |  |  |  |  |
|  | HR | 0.00 | 0.00 | -0.06 | 0.04 | **1.00** |  |  |  |  |  |
|  | RT | 0.10 | -0.06 | -0.01 | 0.05 | -0.09 | **1.00** |  |  |  |  |
|  | TTI 1 | 0.03 | 0.04 | -0.08 | 0.04 | *** 0.27 | 0.05 | **1.00** |  |  |  |
|  | TTI 2 | 0.02 | 0.05 | * -0.12 | 0.04 | *** 0.29 | -0.02 | *** 0.54 | **1.00** |  |  |
|  | AD 1 | 0.05 | 0.07 | -0.03 | -0.03 | * 0.13 | -0.02 | 0.11 | 0.11 | **1.00** |  |
|  | AD 2 | 0.05 | 0.09 | -0.05 | -0.02 | * 0.14 | -0.02 | * 0.11 | 0.10 | *** 0.71 | **1.00** |

**BS2** = blood sampling after behavior tests; **logIgA** = log-transformed immunoglobulin A; **RT** = rectal temperature; **HR** = heart rate; **TTI1** = first observation of tolerance to tactile interaction; **TTI2** = second observation of tolerance to tactile interaction; **AD1** = first observation of avoidance distance; **AD2** = second observation of avoidance distance.

*Significance of Spearman correlation coefficients: *P ≤ 0.05 (highlighted in blue); ****P ≤ 0.001* (highlighted in violet)*.*

**Table S2_3: Correlations between blood and physiological biomarkers at BS1 on blood and physiological biomarkers at BS2** (additional results from the chapter “Effects of blood and physiological biomarkers at BS1 on blood and physiological

biomarkers at BS2”)

| **BS2** | | | | | | | | | | | |
| --- | --- | --- | --- | --- | --- | --- | --- | --- | --- | --- | --- |
| **BS1** |  | Cortisol | Dopamin | Serotonin | logIgA | HR | RT | TTI 1 | TTI 2 | AD 1 | AD 2 |
|  | Cortisol | 0.04 | -0.02 | 0.02 | 0.07 | 0.08 | 0.01 | - | - | - | - |
|  | Dopamin | 0.05 | *** 0.50 | *** 0.28 | 0.00 | * -0.13 | 0.00 | - | - | - | - |
|  | Serotonin | 0.10 | *** 0.32 | *** 0.47 | 0.02 | -0.11 | 0.03 | - | - | - | - |
|  | logIgA | 0.00 | 0.16 | 0.01 | ** 0.21 | 0.05 | 0.02 | - | - | - | - |
|  | HR | 0.06 | 0.08 | 0.09 | 0.00 | *** 0.39 | 0.01 | - | - | - | - |
|  | RT | 0.07 | -0.08 | 0.03 | 0.06 | 0.04 | 0.02 | - | - | - | - |

**BS1** = blood sampling after calving; **BS2** = blood sampling after behavior tests; **logIgA** = log-transformed immunoglobulin A; **RT** = rectal temperature; **HR** = heart rate; **TTI1** = first observation of tolerance to tactile interaction; **TTI2** = second observation of tolerance to tactile interaction; **AD1** = first observation of avoidance distance; **AD2** = second observation of avoidance distance.

*Significance of Spearman correlation coefficients: *P ≤ 0.05 (highlighted in blue); **P ≤ 0.01 (highlighted in green); ***P ≤ 0.001 (highlighted in violet)
